# A draft genome sequence of the rose black spot fungus *Diplocarpon rosae* reveals a high degree of genome duplication

**DOI:** 10.1371/journal.pone.0185310

**Published:** 2017-10-05

**Authors:** Enzo Neu, Jonathan Featherston, Jasper Rees, Thomas Debener

**Affiliations:** 1 Institute for Plant Genetics, Leibniz University Hannover, Hannover, Germany; 2 Agricultural Research Council, Biotechnology Platform, Onderstepoort, Pretoria, South Africa; University of Lausanne, SWITZERLAND

## Abstract

**Background:**

Black spot is one of the most severe and damaging diseases of garden roses. We present the draft genome sequence of its causative agent *Diplocarpon rosae* as a working tool to generate molecular markers and to analyze functional and structural characteristics of this fungus.

**Results:**

The isolate DortE4 was sequenced with 191x coverage of different read types which were assembled into 2457 scaffolds. By evidence supported genome annotation with the MAKER pipeline 14,004 gene models were predicted and transcriptomic data indicated that 88.5% of them are expressed during the early stages of infection. Analyses of k-mer distributions resulted in unexpectedly large genome size estimations between 72.5 and 91.4 Mb, which cannot be attributed to its repeat structure and content of transposable elements alone, factors explaining such differences in other fungal genomes. In contrast, different lines of evidences demonstrate that a huge proportion (approximately 80%) of genes are duplicated, which might indicate a whole genome duplication event. By PCR-RFLP analysis of six paralogous gene pairs of BUSCO orthologs, which are expected to be single copy genes, we could show experimentally that the duplication is not due to technical error and that not all isolates tested possess all of the paralogs.

**Conclusions:**

The presented genome sequence is still a fragmented draft but contains almost the complete gene space. Therefore, it provides a useful working tool to study the interaction of *D*. *rosae* with the host and the influence of a genome duplication outside of the model yeast in the background of a phytopathogen.

## Introduction

Fungal and oomycete pathogens are responsible for the most devastating plant diseases in temperate regions of the world [1, 2]. Many pathogens have developed sophisticated strategies to colonize and exploit their hosts by breaching various lines of defense and by manipulating the defense response of the host [3, 4]. The advent of next generation sequencing technologies has facilitated the sequencing of many fungal genomes, among which the phytopathogenic fungi have particular importance for plant diseases research. The PhytoPath [5] database contains more than 80 genomes of phytopathogenic fungi, which have been sequenced to completion, allowing crucial insights into genomic adaptations to parasitic or hemiparasitic lifestyles [6, 7]. In addition to the assembly of full genomes, draft genomes with more fragmented assemblies are very useful tools in capturing the gene space of a particular species and to identify factors relevant for host pathogen interactions [8, 9, 10].

Most sequenced phytopathogenic fungi have compact nuclear genomes of less than 50 Mb, although in exceptional cases this value can exceed 100 Mb [7].

Phytopathogenic fungi have a number of genomic characteristics that are thought to be adaptations to a parasitic lifestyle. As an example the genomes of hemibiotrophic and necrotrophic parasites encode more enzymes involved in the breakdown of complex carbohydrates than other fungal groups [11, 12]. Common to all phytopathogens are genes encoding secreted effector proteins, which are involved in the invasion process [6, 12]. As some of these effectors, or the changes they induce in the host metabolism, are recognized by the innate immune system of plants, their study is of considerable interest to both basic and applied research. Furthermore, these data can be utilized for comparative studies among diverse fungal taxa to unravel the evolutionary dynamics of adaptations involved in host-plant interactions.

Rose black spot is caused by the hemibiotrophic ascomycete *Diplocarpon rosae* (its anamorph is *Marssonina rosae*) and is one of the most damaging diseases of garden roses. Due to the world-wide trade of rose cultivars the pathogen has spread over all temperate regions of the world. The damage caused by this disease is not only indirect, due to the loss of aesthetic value of the commercial product, but also direct by weakening plants to the point of killing extremely susceptible genotypes. Propagation of the pathogen is mainly due to asexually propagated conidia spread by water and direct contact [13]. The infection starts with the germination of bicellular conidia, the formation of a germ tube and penetration of the cuticle via appressoria. During the early biotrophic stage the fungus invades the host tissue with mycelia that develop intracellular haustoria for the extraction of nutrients. This is followed by a mixed biotrophic/necrotrophic phase resulting in some tissue damage [14]. The rose black spot interaction is one of the best studied plant pathogen interactions for cultivated roses [15]. To date up to 11 pathogenic races differentiated on different sets of host plants have been characterized by various authors [15, 16] and the interaction of host and pathogen has been studied by histological and biochemical methods [14, 17, 18]. On the host side several studies addressed host resistance and a number of R-genes (resistance genes) were characterized [19, 20, 21], one of which was characterized as a TNL type resistance gene which mediates resistance against different isolates of the pathogen including DortE4 [22, 23, 24]. An interesting aspect of the pathogen biology relates to observations that indicate a low mobility of new genetic variants within and between host populations most probably due to the spread of conidia via splash water [25]. This will make disease resistance management strategies based on single R-genes interacting with single avirulence (Avr) genes more useful compared to pathosystems with extremely mobile pathogens such as powdery mildews [15].

In this study we present the draft genome sequences of the *D*. *rosae* isolate DortE4, which is one of the prevalent races interacting with the well-studied Rdr1 resistance locus. This genomic sequence will serve as a working tool for the analysis of the gene space of this plant pathogen and in particular its pathogenic features. The draft can be also used as a tool to develop genetic markers for studying the population biology of the fungus. In addition to the functional information of the genome we will also analyze the genome structure and compare it to other fungi and other *D*. *rosae* isolates.

## Results and discussion

### Genome assembly

A total of 1.3 million 454 and 166.7 million Illumina paired end reads were combined to assemble the *D*. *rosae* genome with approximately 191-fold coverage (Table 1). These reads were assembled to contigs which were connected to 2618 scaffolds (>500 bp) with an N50 size of 243.6 kb and a total assembly length of 66.6 Mb (Table 2). These data are comparable to other fungal draft genomes [8, 9, 26] but especially the N50 value and the size of the longest scaffold (approximately 1 Mb) are outstandingly long and much larger than for others assemblies generated using similar sequence datasets. Nevertheless the genome is still very fragmented even if one takes only larger scaffolds with more than 500 bp into account.

**Table 1:** Sequencing statistics.

Read type	No. of reads[M]	No. of bases [Gb]	Coverage
**Roche 454**	1.3	0.53	6x
**Illumina (short insert)**	40.1	3.19	37x
**Illumina (long mate pair)**	126.8	12.53	147x

**Table 2:** Key data of the *D*. *rosae* assembly.

**Number of scaffolds ≥ 500 bp**	**2457**
**Longest scaffold**	974.8 kb
Total length*	66.6 Mb
N50*	243.6 kb
L50*	84
GC-content*	47.64%
Fraction of ambiguous bases*	4.45%

*Values calculated with scaffolds larger than 500 bp.

### Determination of the genome size

**Fig 1:**
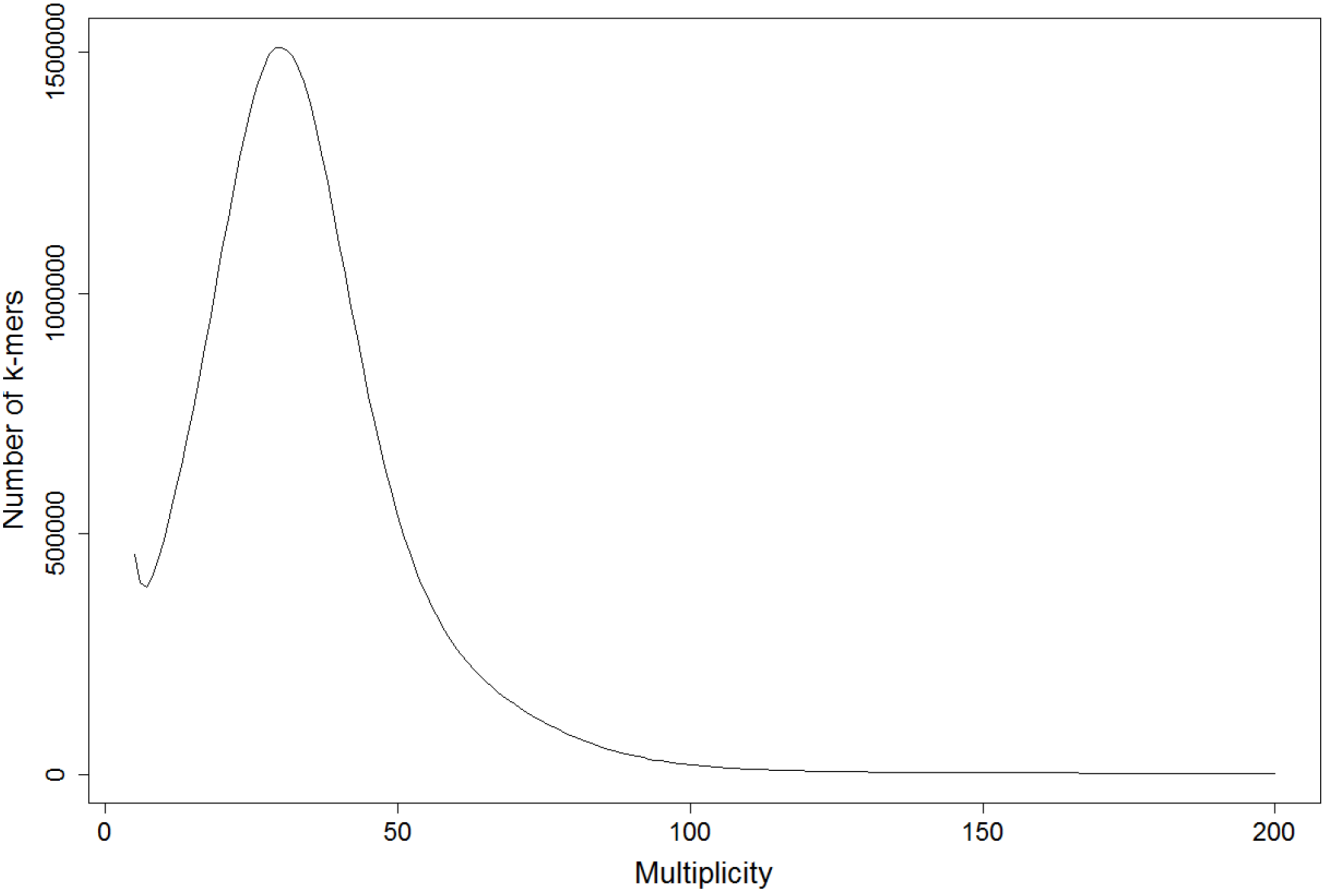
k-mer distribution based on the small insert Illumina library. The plot displays the number of k-mers (size 17) generated from the reads (y-axis) that occur with a given multiplicity (x-axis). The peak represents the mean k-mer depth. K-mers with extremely low frequency (<5) are not displayed and are considered to contain sequencing errors.

Flow cytometry is one of the most effective methods for the estimation of the genome size of eukaryotes and has largely replaced cytological methods. However, the adaptation of this technique to the analysis of fungal genome size is in some cases a challenging task [27, 28]. So far, we were unsuccessful to apply flow cytometry to *D*. *rosae* because we were not able to isolate sufficiently pure and intact nuclei. Therefore, we estimated the genome size with three different approaches based on the k-mer distribution of the reads resulting from the small insert library. An example of a k-mer distribution is depicted in Fig 1, more details are given in the supplemental file S1 File. The plot shows a clear unimodal distribution with maximum abundance at a k-mer coverage of 30.

The first two approaches of Li and colleagues applied for the giant panda genome [29] and Liu and colleagues for *Brassica oleracea* [30] are similar because they estimate the sequencing depth based on the k-mer coverage and calculate the genome size from this sequencing depth. Both approaches resulted in similar genome size estimations. Depending on the used k-mer size the genome size ranges from 84.99 Mb to 91.36 Mb with the approach of Li *et al*. and 83.61 to 88.6 Mb with the approach of Liu *et al*. (S1 File).

For the third approach the GenomeScope webserver [31] was used, which fits a mixture model of negative binominal model terms based on the k-mer distribution. The estimations with this software are smaller than those calculated with the other two approaches and range from 72.52 Mb to 73.53 Mb. One problem with this approach is that the software fits the model based on four peaks, which it tries to identify in the distribution. In the given data these peaks are hardly visible (S1 File), which influences the model and makes the results more erroneous.

The fungal genome size database [32] contains the data of more than 1300 ascomycetes including values derived from assembly sizes of complete genome sequencing, and from experimental methods like flow cytometry or pulse field gel electrophoresis. Taking only the experimental data into account, the mean genome size for Ascomycota is 49.4 Mb. Unfortunately, only very few plant pathogenic fungi are included in these data. One of them is *Sclerotinia sclerotiorum* which belongs to the same order as *D*. *rosae*. Depending on the method used, its genome size is determined to be between 43.5 Mb [33] and 53.77 Mb [34]. Both values are much smaller than those calculated for *D*. *rosae*.

More data are available in the PhytoPath database [5] which collects genomic sequences and the corresponding assembly length of phytopathogens. According to this database the model organism *Magnaporthe oryzae* has an expected genome size of 41 Mb and the fully sequenced species *Botrytis cinerea* and *Marssonina brunnea*, which are closely related to *D*. *rosae*, with 42 Mb and 52 Mb have much smaller genomes than black spot fungus, even compared to the assembly presented here (Table 2). However the database also contains sequences of fungal pathogens which have almost the same or even larger genome sizes than *D*. *rosae*. The ascomycetes *Pseudocercospora fijiensis*, *Blumeria graminis* and *Verticillium longisporum* have comparable or larger genome assemblies with 73.7 Mb, 87.9 Mb and 100.5 Mb respectively. Also the *Puccinia* species, belonging to the Basidiomycota phylum, have extremely large genome sizes that range from 81.6 to 163 Mb. Despite this broad range of genome sizes, the calculated genome size of *Diplocarpon rosae* seems unexpectedly large if compared to close relatives and the majority of other phytopathogenic ascomycetes.

### Annotation of the draft genome

For annotation the MAKER pipeline [35] was used with additional supporting evidence from other published closely related species and our own transcriptomic data. Table 3 summarizes the key data from the genome annotation. The number of *de novo* genes predicted ranges from 16,304 with Augustus [36] to 19,172 with the self-trained tool GeneMark-ES [37]. As a result of the MAKER pipeline, 14,004 high quality gene models were annotated on the scaffolds. The gene models have an average length of 1854 bp (S2 File), and contain an average of 3.4 exons with a mean length of 487 bp and a mean intron length of 94 bp. The predicted gene and intron length is consistent with genes found in other ascomycete genomes [38]. The other values are slightly higher than expected but as Galagan *et al*. and Mohanta and Bae showed in their reviews fungal genomes are extremely variable so that the number of genes can range from less than 6,000 to more than 27,000 and there are also examples where the mean number of exons per gene exceeds 4 [38, 39].

**Table 3:** Key data of the annotation pipeline.

**Gene annotation with the MAKER pipeline**	
**Prediction tool**	**Number of predicted sequences**
Augustus	16,304
GeneMark-ES	19,172
SNAP	16,931
MAKER High quality gene annotations	14,004
**Functional annotation with Blast2Go**	
**Annotation step**	**Number of annotated sequences**
Blast	13,562
Mappings (GO term assignment)	11,666
B2G: high quality annotations	10,043
InterProScan	10,839

Automatic functional annotation of the 14,004 gene models was then performed using Blast2GO [40], which assigns a functional description to sequences based on the top 20 Blast matches. In addition to this, the program also assigns GO-terms and performs an InterProScan [41] to provide additional characterization.

With an analysis using Blastx 96.8% of the predicted genes have a statistically significant (E-value ≤ 1e-10) match in the NCBI-NR database (S2 File). More than 70% of these matches originate from the fungus *M*. *brunnea* (S2 File) again indicating their close relationship. Most of Blast matches display similarities of more than 50% to the target sequences with an average value of 73.2% (S3 File). GO terms were assigned to 11,666 of the sequences and only 1623 of these GO annotations did not pass the internal quality control of Blast2Go. In this way more than 50,000 high quality GO annotations were assigned to 10,043 sequences. In addition to the GO-annotation, 2460 genes were assigned to an ENZYME EC number [42] and the InterProScan linked functional information for 10,839 gene models (S3 File).

### Transcriptomic analysis of the predicted gene space

With a combination of the MACE (Massive Analysis of cDNA Ends) and RNA-Seq a transcriptomic dataset was generated covering the first stages of the compatible interaction between black spot and roses to analyze transcription of the predicted black spot genes. The data comprises three time points (0, 24, 72 hpi) represented by three (MACE) to six (MACE and RNA-Seq) datasets each set based on independent inoculation experiments of DortE4 with the susceptible variety “Pariser Charme”. These sequence data were mapped to the annotated genomes to analyze the portion of predicted transcripts expressed over the first three days of the infection process.

**Fig 2:**
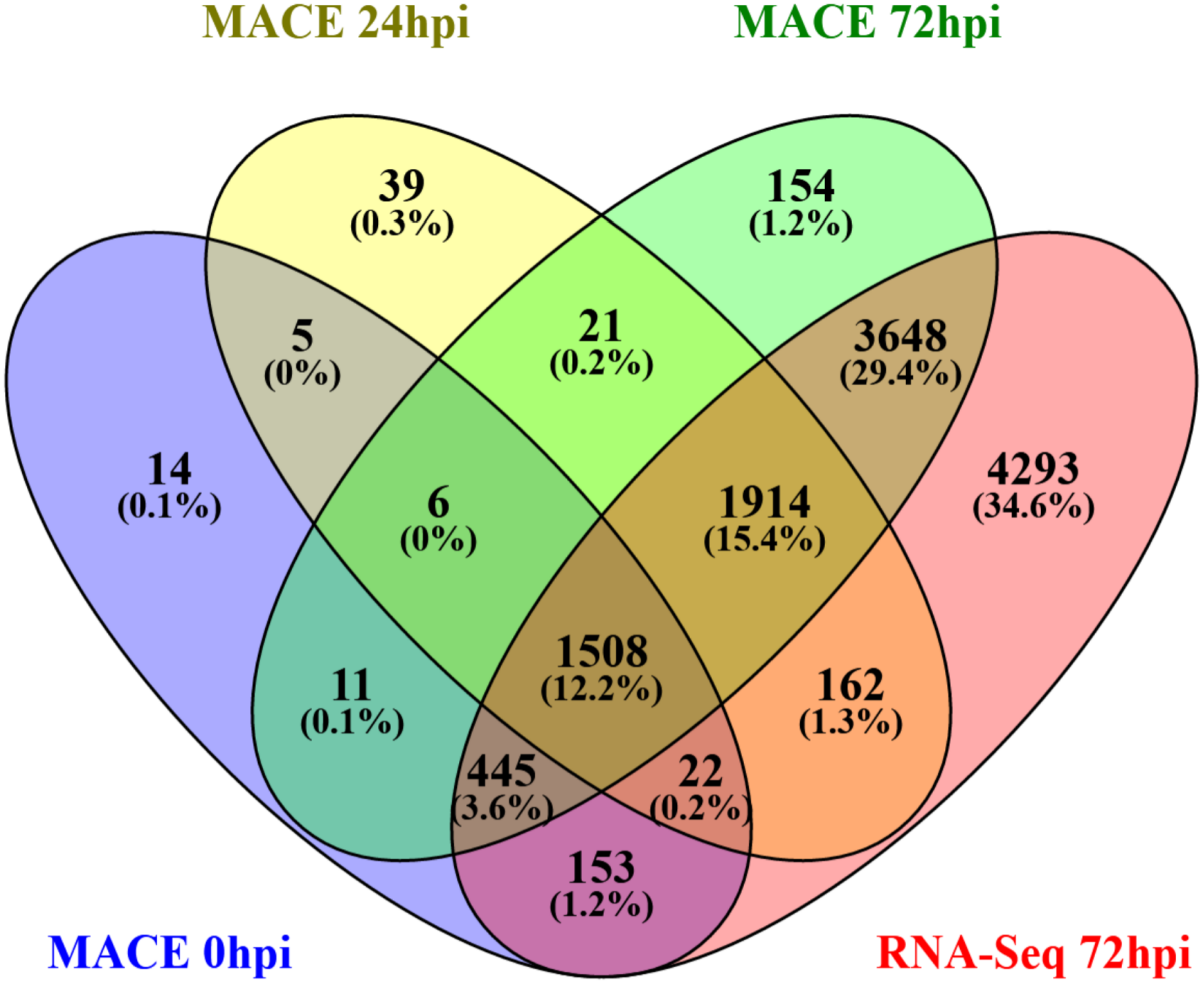
Venn diagram of expressed genes during early stages of the infection process (0, 24, 72 hpi). Three independent inoculation experiments were performed and genes are considered to be expressed at a time point, if they show expression in at least two of the three experiments. MACE and RNA-Seq data for 72 hpi were generated from the same samples.

A total of 12,396 (88.5%) of the predicted genes were represented by expressed transcripts, with the majority (8095) of these genes exclusively detectable three days after inoculation (Fig 2, S4 File). As our data only cover the first phase of the fungal development, including the formation of haustoria while other developmental stages of the fungus such as the formation of plectenchymatic tissues, acervuli and conidia as well as the sexual stages were not included, so it is not surprising that not all genes were found to be expressed. Another reason for undetected transcripts is the fact that in total only 1.5 million MACE and 2.4 million RNA-Seq reads matched the genome of *Diplocarpon*. The majority of the sequenced reads originated from the rose transcriptome because at early stages of infection the fungal biomass is comparable small to the amount of plant tissue. However, at later stages where the growing fungal mycelium has increased in biomass, many more transcripts could be detected. The fact that the majority of the transcripts occur three days after inoculation is most probably due to this effect and the development of additional/new organs like e.g. haustoria which are not detectable until 24 h after inoculation.

Another interesting point is that with the RNA-Seq data 12,145 transcripts were detected at 72 hpi whereas with the MACE data only 7,407 (Fig 2). This might be due to the MACE procedure where only the 3’ ends, especially UTR regions of the mRNA molecules, were sequenced. But these regions are especially hard to predict by the *ab initio* gene prediction tools, indicating that the current annotation might underestimate the UTR regions of the genes. At the same time, there are transcripts that were only detected by the MACE data. These might be low abundance genes, which can be better detected by MACE than RNA-Seq [43].

### Pathogenic features of the genome

To get a better insight into the putative functions of the annotated sequences in the pathogenic process, two databases (the Phi-base and the CAZy database) were used to gather additional information. The Phi-base (pathogen-host interaction database) [44, 45] contains virulence, pathogenicity and effector genes of fungi, oomycetes and bacteria. The entries are categorized based on information about the influence of mutagenesis experiments on the virulence of the pathogens. Using Blastp (E-value ≤ 1e-10) a total of 3683 *D*. *rosae* predicted genes matched sequences in the Phi-database. Of these 17 are classified as known effectors, 346 showed a loss of pathogenicity in mutagenesis experiments and 1530 a reduction of virulence (S5 File).

58% of these sequences are detected by the MACE analysis and 90% by the RNA-Seq data, indicating that they are expressed during the early stages of the infection. Of particular interest for further analysis are the 15 expressed homologs of known effectors because they can give a closer insight into the interaction between the pathogen with its host. Finding functional effectors can give information about how the pathogen modulates the host immune response or acquires nutrients from it. They can also be useful tools for finding new R-genes and to decode their mode of action. But also the other virulence factors can give valuable information about the pathogenic strategy of a hemibiotrophic lifestyle.

The CAZy database [46] was used to detect groups of genes that code for carbohydrate degrading, modifying and synthesizing enzymes. This includes cell wall degrading enzymes (CWDEs) like cellulases, glucanases, xylanases, pectin lyases and other hydrolytic enzymes. With the dbCAN webserver [47] 724 of the predicted genes were assigned to protein families from the CAZy database (S6 File) including 285 glycoside hydrolases (GH) from 49 families, 129 carbohydrate esterases (CE), 168 glycosyl transferase (GT), 32 polysaccharide lyases (PL), 38 sequences carrying a carbohydrate-binding module (CBM) and 104 with auxiliary activity (AA). The last family contains among others lignin peroxidases (AA2), cellobiose dehydrogenases (AA3) and lytic polysaccharide monooxygenases (AA9) [48]. Kubicek and colleagues compared the CWDE content of 35 saprophytic, necrotrophic and hemibiotrophic fungi [11]. In general the content of CWDEs in the *D*. *rosae* genome corresponds with other hemibiotrophic fungi, but some features like the large number of cellulases, especially of the family GH5, and many pectin degrading enzymes (GH28, GH78, PL1, PL3, PL4, PL9 and PL11) match more with necrotrophic fungi. It is also noticeable that the genome contains fewer genes for hemicellulolytic enzymes than other fungi. This might be due to differences in the cell wall composition of roses or may just be another reflection of the extreme diversity of fungal genomes.

Of particular interest are the five sequences, which are assigned to the LysM-family (CMB50, IPR018392). This protein class might contain effector proteins. The Ecp6 protein of *Cladosporium fulvum* has been shown to interfere with the chitin induced defense response due to the chitin binding function of this domain [49]. Similar functions are reported for LysM-proteins from *M*. *brunnea*. It is shown that LysM-proteins of this fungus can interact with chitin in a way similar to Ecp6. In addition, expression of two different fungal LysM-protein with plant secretion signals in *A*. *thaliana* led to a reduced induction of *PDF1*.*2* expression in response to chitin, indicating a reduced chitin dependent defense response [50]. Therefore, the five expressed LysM-genes (S4 File) are useful starting points for further analysis and can complement the genes identified from the PHI-database.

### Repeat structure of the *Diplocarpon* genome

An analysis of the repetitive fraction of the genome (Table 4) revealed an unexpectedly small fraction (15%) of repeats in the *Diplocarpon* genome in comparison to its large predicted genome size. In general this amount of repetitive and transposable elements is large compared to other fungi. The reference genome of the model fungus *M*. *oryzae* contains only 9.7% of repetitive elements and the assemblies of the related fungi *S*. *sclerotiorum* and *B*. *cinerea* contain only 7% respectively less than 1%. However, these genomes are all much smaller than that of *D*. *rosae*. In many reported cases genome enlargement is due to an expansion of the noncoding DNA especially transposable elements (TEs) and repeats [51, 52]. The genome of *Blumeria graminis* for example has a size of around 120 Mb and consists of 64% TEs [53]. The same was observed for the genome of *Tuber melanosporum* with a size of 125 Mb and a TE portion of 58% [54]. Even the smaller genome (52 Mb) of the closeted sequenced relative *M*. *brunnea* has a much higher repeat content of 42% [55]. There is a possibility that in the presented sequence some TEs collapsed during the assembly, which might be an explanation for the difference between the assembly length (66.6 Mb) and the predicted genome size (73–91 Mb).

**Table 4:** Distribution of transposable elements and repeats in the genome sequence.

Type	Number	Length [bp]	Percent of the assembled genome sequence
**SINEs**	0	0	0
**LINEs**	163	164,700	0.24
**LTR elements**	8428	4,812,228	7.10
**DNA transposons**	562	195,740	0.29
**Rolling-circles**	140	114,992	0.16
**Unclassified**	11309	3,748,693	5.53
**Simple repeats**	22241	902,353	1.33
**Low complexity**	2545	127,064	0.19

SINE: short interspersed nuclear element

LINE: long interspersed nuclear element

LTR: long terminal repeat

A total of 7.8% of the genome assembly is composed of mobile genetic elements with LTR elements representing the largest group (7.10%) (Table 4). All LTR elements belong to the Copia or Gypsy family (S7 File) which is not surprising, because these families are the most often reported types of transposable elements in fungi [51]. The other class of retrotransposons, the non-LTR elements (SINEs and LINEs), represent only 0.24% of the assembled sequence in which no SINE elements were detected. DNA transposons and rolling cycle elements tend to play a secondary role with less than 0.5% of the genome assembly each. Those results reflect the above mentioned general trend that the amount of transposable elements is larger than that of other fungal genomes but not as large as expected. For the genome of *M*. *oryzae* only 3.4% of LTR retrotransposons were reported, for *S*. *sclerotiorum* and *B*. *cinerea* less than 1% [55]. Much larger amounts of these elements were detected in the genome of *M*. *brunnea* and *B*. *graminis* with approximately 26% respectively 12.4%. For non-LTR retroposons and DNA transposons the picture is different because out of the mentioned fungi only *B*. *cinerea* contains smaller amount of these elements than the analyzed assembly. All other sequences contain between 1.1% and 1.9% of these elements. *B*. *graminis* is an exception here because with more than 20% an outstanding amount of non-LTR elements is reported in its genome [51, 53]. The trend for the unknown repetitive elements as well as the more undefined simple repeats and low complexity regions is that these elements occur in higher rates in the *D*. *rosae* sequence compared to other fungal genomes but not in rates as high as in *M*. *brunnea* and *B*. *graminis*.

Altogether, those findings indicate that the content of repetitive elements is higher in the presented assembly than in other fungal genomes but the large genome size cannot be explained by these elements alone.

Apart from the relevance of the repeat content for the genome structure, this information can also be used to generate simple sequence repeat (SSR) markers which can used in studies about the population biology of *Diplocarpon*. Therefore, the genome sequence of DortE4 and related plant pathogenic fungi was screened with the SSR locator tool [56]. The results, (Table 5) indicate that the *D*. *rosae* genome contains a total of 8242 SSR motifs, which is less than *M*. *brunnea*. This corresponds to the lower repeat content as mentioned before. Interestingly *M*. *oryzae*, which contains only 9.7% repetitive sequences in its 41 Mb sized genome, has almost the same content of SSRs as *D*. *rosae*. This indicates that repeat structure and SSR content do not correlate well in these organisms.

**Table 5:** SSR content of the genome.

Species	Assembly size [Mb]	Mono	Di	Tri	Tetra	Penta	Hexa	Hepta	Octa
***D*. *rosae***	66.6	6150	1629	350	58	21	16	6	12
***M*. *brunnea***	52	10618	1229	494	196	66	63	27	36
***B*. *cinerea***	41.2	3024	545	162	89	24	33	25	20
***S*. *sclerotiorum***	38.5	2403	112	88	68	36	64	53	23
***M*. *oryzae***	41	8375	277	102	12	3	19	3	5

Neither is there a correlation between the genome size and the number of SSR motifs, as can be seen by comparing the genome of *M*. *brunnea* with that of *D*. *rosae*. The genome of *M*. *brunnea* is smaller than that of *D*. *rosae*, but contains more SSRs. The same pattern emerges when comparing the genomes of *B*. *cinerea* and *S*. *sclerotiorum*, which have comparable genome sizes of 42 Mb and 38 Mb respectively but huge differences in SSR content. The same was observed in a study by Karaoglu, Lee and Meyer [57], which examined SSR motif abundance in nine different fungal genomes of very diverse sizes. They found no correlation between the genome size and SSR content.

### Coverage of the gene space based on BUSCO analysis

To estimate if the genome assembly, as well as the predicted transcriptome, contains the whole gene space of *Diplocarpon*, an analysis was performed with the BUSCO pipeline [58]. This pipeline examines the presence of single-copy orthologs, which are conserved between almost all species within one phylogenetic clade. For fungi this dataset comprises 1438 genes. For the genomic sequence of *D*. *rosae*, 96.3% of the BUSCO orthologs were detected as complete sequences, 2.8% were fragmented and less than 1% are missing (Table 6). Almost the same results were found for the predicted transcriptome with 96.5% full length sequences and 1.5% fragmented sequences. Only 1.9% of the orthologs are missing in the predicted transcriptome, although they are detectable at the genomic level but with no gene model predicted. Therefore, the gene prediction might lead to a slight underestimation of the gene space.

**Table 6:** Comparison of BUSCO results of the *D*. *rosae* sequence and other fungi.

	Complete BUSCOs	Complete single-copy BUSCOs	Complete duplicated BUSCOs	Fragmented BUSCOs	Missing BUSCOs
***D*. *rosae***	1385	277	1108	41	12
**[%]**	96.3	19.3	77	2.8	0.8
*D*. *rosae* trans.*	1388	322	1066	22	28
**[%]**	96.5	22.3	74	1.5	1.9
***M*. *brunnea***	1412	1337	75	20	6
**[%]**	98.2	93.0	5.2	1.4	0.4
***B*. *cinerea***	1423	1340	83	12	3
**[%]**	98.9	93.2	5.7	0.8	0.2
***S*. *sclerotiorum***	1387	1301	86	42	9
**[%]**	96.5	90.5	5.9	2.9	0.6
***M*. *oryzae***	1418	1338	80	10	10
**[%]**	98.6	93.0	5.5	0.7	0.7
*S*. pastorianus**	1372	488	884	45	21
**[%]**	95.4	33.9	61.5	3.1	
***A*. *macrogynus*** **	1080	316	764	178	180
**[%]**	75.1	22.0	53.1	12.4	12.5

*predicted transcriptome of *D*. *rosae*,

**sequences of diploid fungi

Comparing these results to the BUSCO analysis of other sequenced fungal genomes indicates that our draft genome covers the gene space almost as completely as the fully sequenced genomes of *M*. *brunnea* [55], *B*. *cinerea*, *S*. *sclerotiorum* [59] and the model organism *M*. *oryzae* [60], which contain between 96.5% and 98.9% of the BUSCO orthologs as full length sequence and between 0.7% and 2.7% as fragmented sequences.

However, the genomes differ in the number of orthologs found in multiple copies that normally occur as single copies. In total, 77% of the complete BUSCO genes in the *D*. *rosae* genome were duplicated, whereas other plant pathogen genomes analyzed contain less than 6% duplicated orthologs. Simao *et al*. presented results of their pipeline for 15 fungal genomes and neither of these showed a duplication rate over 11% [58]. This is a strong indication that there is duplication of large parts or the whole genome of *D*. *rosae*. To examine this hypothesis we tested the genome sequences of two polyploid fungi, the Blastocladiomycete *Allomyces macrogynus* (ATCC 38327) (Broad Institute, Acc. no. ACDU01000000) and the ascomycete *Saccharomyces pastorianus* (CBS 1513) [61] with the BUSCO pipeline. The numbers of duplicated genes are with 53.1% and 61.5% comparable to the results of our genome sequence (Table 6) and support the hypothesis that a genome duplication has occurred relatively recently in *D*. *rosae*.

### Analysis of the duplicated portion of the genome

To rule out technical factors, such as assembly errors, we analyzed the putatively duplicated portion of the black spot genome in more detail. Based on the BUSCO analysis we selected those gene predictions that are BUSCO orthologs and that possess paralogs in the genomic sequence (S8 File). 95.4% of the duplicated BUSCO orthologs occur in pairs, only 3.5% in three copies and less than 1.1% are present at four or five copies in the genome. This is a clear indicator of a single genome duplication. At the same time one would expect a larger diversity of copy numbers if the result was due to an error in the assembly.

**Fig 3:**
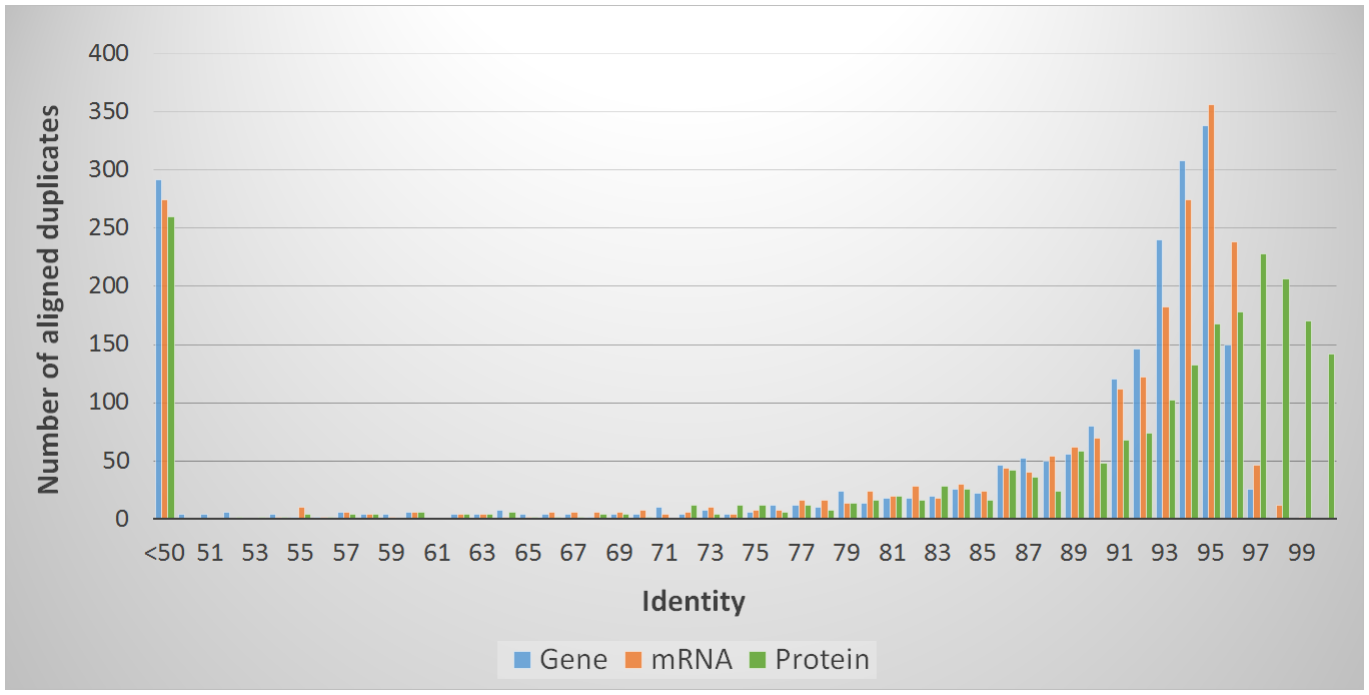
Identity values of *D*. *rosae* paralogs. Histogram of identity scores generated by global alignment of all duplicated BUSCO orthologs in the *D*. *rosae* assembly. The gene alignments include intron sequences.

Another indicator of sequencing or assembly errors would be a high level of sequence similarity of the duplicated sequences in a haploid genome, because they should represent identical sequences at different locations in the assembly. To determine the degree of polymorphism between the paralogs, we performed a global multiple sequence alignment with the mafft tool [62] on the gene, mRNA and protein level with the predicted sequences and calculated an identity matrix based on these data. Fig 3 illustrates the identity scores of the best pairings (S9 File) as a histogram. The majority of duplications share a high degree of identity (more than 90%), on all three levels in the global alignment. The diversity far exceeds the amount of expected sequencing errors, which could lead to mistakes and duplications in the assembly.

An additional line of evidence comes from the transcriptome data. In more than 97% of the cases both paralogs showed expression for at least one time-point (S8 File). In less than 1% of the cases none of the paralogs shows expression and in 2.3% only one of the duplicates is expressed. If the paralogs were just a product of an erroneous assembly the number of pairs which show expression in only one of the sequences would be much higher because the mapping parameters used were very stringent.

**Fig 4:**
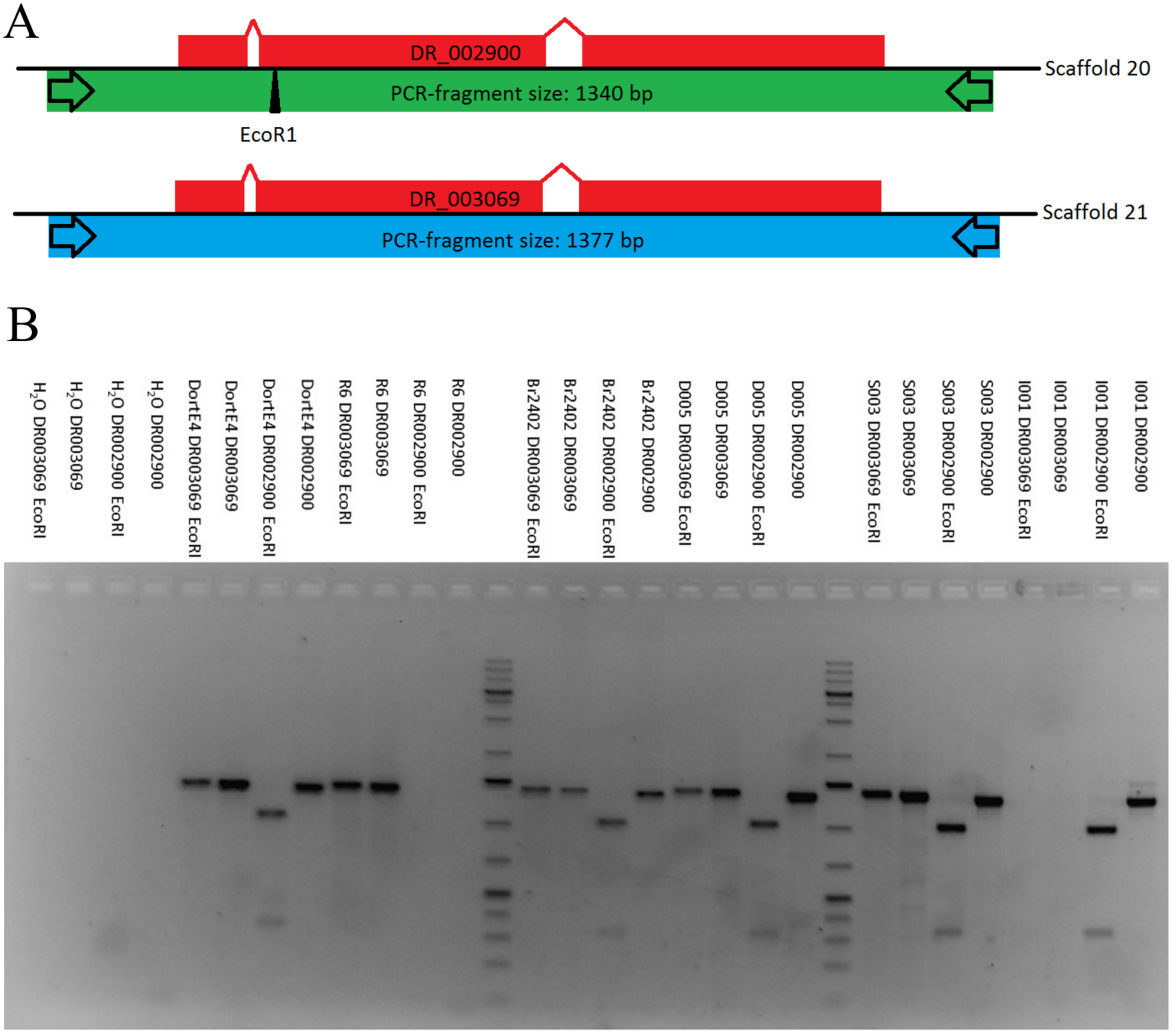
PCR-RFLP analysis of pairs of BUSCO orthologs. A: Schematic display of the method. Specific primers were developed that distinguish between the two very similar paralogs. As second distinctive feature only one of the two PCR fragments contains an EcoRI or DraI restriction site. B: Gel image of a PCR-RFPL analysis of the paralogous sequences DR002900 and DR003069 with six different isolates. Ladder: 1 KB plus (Thermo Fisher, Waltham, USA).

Besides these indictors, which are based on the sequence itself, we developed a PCR-RFLP system to confirm the presence of six paralogous gene pairs (Fig 4A). We designed copy specific PCR primers and tested them on DNA from conidia of the DortE4 isolate which were washed from leaf material, to exclude the possibility that the duplications were due to artificial *in vitro* culture. Differences of restriction sites within the fragments were used as additional evidence that two different gene loci were amplified. For DortE4 all primer pairs produced fragments of the expected sizes and restriction patterns (Fig 4B, Table 7, S10 File). This confirms the results of the BUSCO analysis and effectively demonstrates experimentally that the genome of the isolate DortE4 contains a large proportion of duplication.

**Table 7:** PCR-RFLP results of six pairs of paralogs tested with six different *D*. *rosae* isolates.

BUSCO-ID	*D*. *rosae* paralogs	DortE4	R6	I001	S003	D005	Br2402
**BUSCOfEOG7D2FR2**	DR005870	+	-	+	-	+	+
	DR011096	+	+	-	-	+	+
**BUSCOfEOG7K9KD3**	DR004273	**+**	**+**	**-**	**+**	**+**	**+**
	DR002774	**+**	**+**	**+**	**+**	**+**	**+**
**BUSCOfEOG7MD52Z**	DR002315	**+**	**-**	**+**	**+**	**+**	**+**
	DR001421	**+**	**+**	**-**	**+**	**+**	**+**
**BUSCOfEOG7R83CB**	DR002947	**+**	**+**	**+**	**+**	**+**	**+**
	DR003022	**+**	**+**	**-**	**+**	**+**	**+**
**BUSCOfEOG783N6S**	DR002900	**+**	**-**	**+**	**+**	**+**	**+**
	DR003069	**+**	**+**	**-**	**+**	**+**	**+**
**BUSCOfEOG7DRJFG**	DR008457	**+**	**+**	**-**	**+**	**+**	**+**
	DR005710	**+**	**-**	**-**	**+**	**+**	**-**

**(+)** indicates the presence of the fragment as well as the expected restriction pattern

**(-)** indicates the absence of a PCR fragment

In the next step five additional isolates were tested with the PCR-RFLP system with contrasting results concerning the individual gene duplications (Table 7, Fig 4B). Only D005 contained all duplicates. S003 and Br2402 differed for one of the paralogous pairs. The isolates R6 and I001 show a difference in almost all duplicated pairs but interestingly they do not share the same pattern of differential loss even though they belong to the same race (race seven) [16]. This might indicate that the duplication is already in the state of reduction by gene loss as has been reported for yeast and other fungi [63, 64, 65]. Due to the fact that the tested DNAs originated from conidia isolated from leaves and not from *in vitro* material, the possibility that the inconsistencies in the duplication pattern are the result of stress induced spontaneous duplications can be ruled out. Interestingly, the pattern of gene loss does not appear to be race specific indicating that it might not influence the effector content of fungi but more analyses are needed to clarify that.

**Fig 5:**
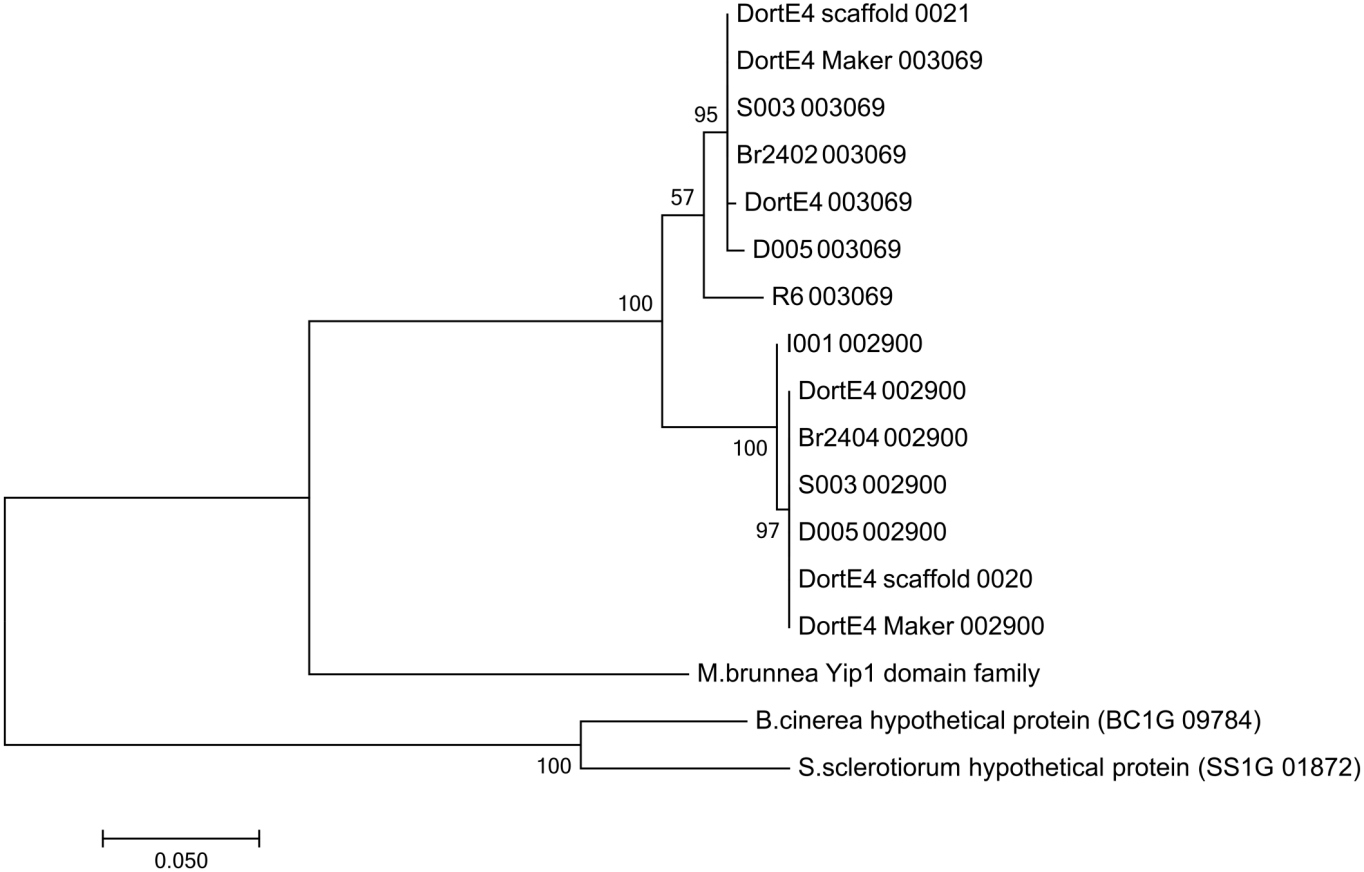
Phylogenetic tree of the paralogous sequences DR002900 and DR003069. The tree contains sequences of six *D*. *rosae* isolates, the predicted mRNA sequences of the two genes, the genomic region of the scaffolds between the primer binding sites and the corresponding orthologs from *M*. *brunnea*, *B*. *cinerea* and *S*. *sclerotiorum*. The tree was generated with the maximum likelihood method. The values on the branches refer to a bootstrap test (500 replicates).

The difference between R6 and I001 is also visible in the phylogenetic analysis regarding the paralogous pair DR002900 and DR003069, which were analyzed in more detail with Sanger sequencing of all 10 PCR products (duplicated BUSCO genes from four isolates where I001 and R6 only amplified one of the paralogs). As it can be seen in the maximum likelihood tree (Fig 5, S11 File) all sequences of the two different genes cluster together with the predicted gene models and the corresponding sequences of the genomic scaffold in two distinct clades, proving again the existence of two distinct genes in the *D*. *rosae* genome where there is only one in the genomes of the related fungi *M*. *brunnea*, *B*. *cinerea* and *S*. *sclerotiorum*. Within these clades I001 and R6 are separated from the other isolates indicating that their sequences contain more polymorphisms than the others.

**Fig 6:**
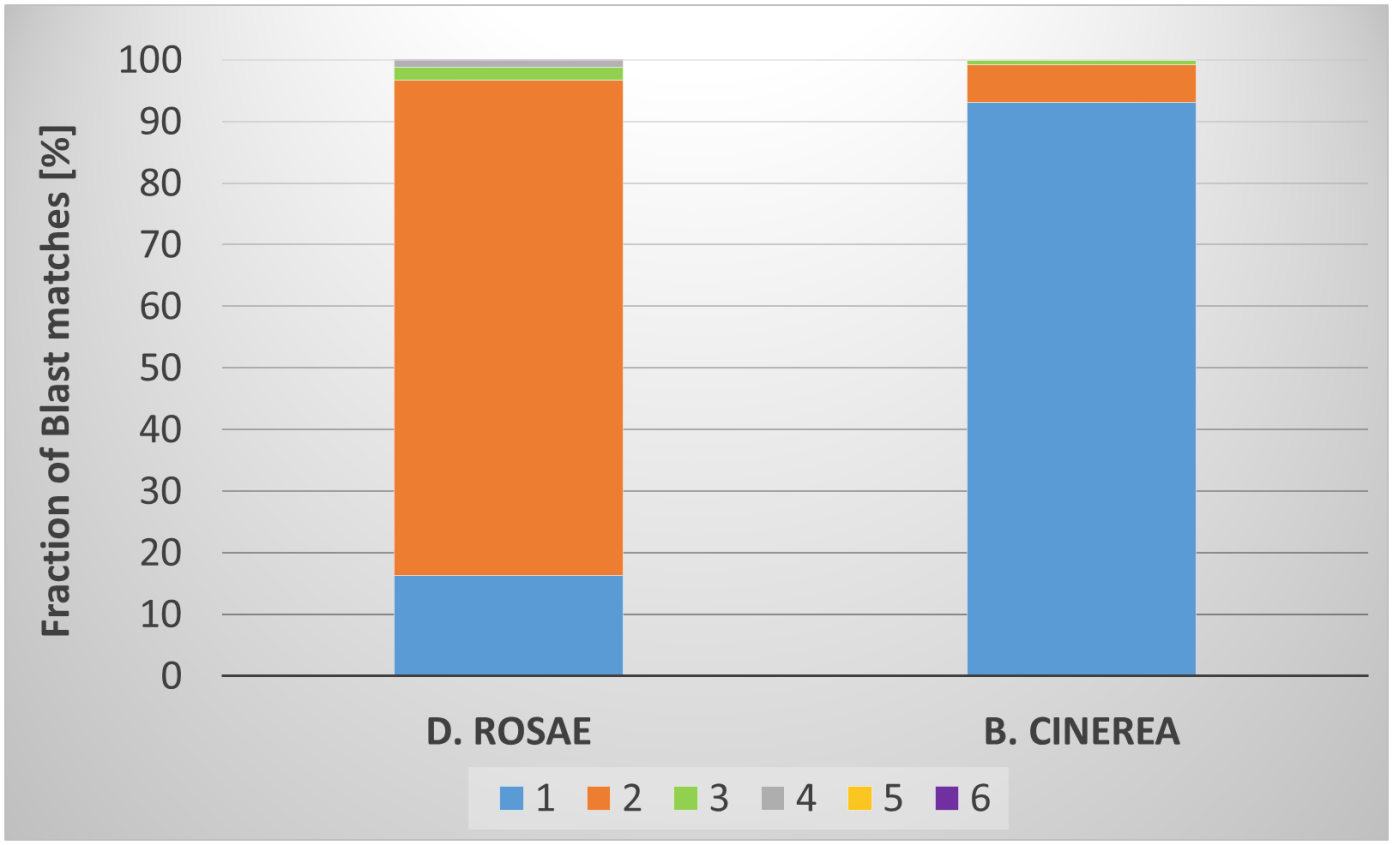
Comparison of the percentage of Blastp matches with different numbers of matches to the same reference sequence between *D*. *rosae* and *B*. *cinerea*. The portion of the *M*. *Brunnea* proteome that had no second match in a Blast (E-value cutoff e-100) of the proteome against itself was the reference for the Blastp search. The cutoff for the Blastp of the related species was set to e-100. The different colors refer to the number of Blast matches to the same *M*. *Brunnea* reference sequence.

To get an idea about the extent of the duplication we compared the predicted proteome of *D*. *rosae* with its relative *M*. *brunnea* using Blastp. To reduce the influence of gene families we used only those protein sequences of the *M*. *Brunnea* proteome that do not match other sequences of the proteome than itself (E-value cutoff e-100). These 8402 sequences were used as a reference for a Blastp search (E-value cutoff e-100) with the predicted *D*. *rosae* proteome and the proteome of *B*. *cinerea* (S12 File). The number of Blast matches differs between the species. 5177 of the *M*. *brunnea* sequences had between one and six matches with sequences from *D*. *rosea*. Less matches were found between *M*. *bruunea* and *B*. *cinerea*. 3724 *M*. *Brunnea* sequences had up to four matches to the *Botrytis* proteome. This difference is not surprising because the relationship between *D*. *rosae* and *M*. *brunnea* is much closer than the relation to *B*. *cinerea*. All three species belong to the same order but different families. The most important point in this comparison is depicted in Fig 6, where the proportion of blast matches that have different numbers of matches to the same reference sequence is shown. These results indicate that approximately 80% of the *M*. *brunnea* sequences had two matches to the *D*. *rosae* proteome and only 16.4% had only one match. The results of the Blast with *B*. *cinerea* show the complete opposite. Here 93% of the *M*. *brunnea* sequences had one hit in the *Botrytis* proteome and only a small fraction of 6.3% had two matches. These results correspond with the BUSCO results. Both analyses indicate a single duplication event in which more than three-fourth of the *D*. *rosae* proteome is duplicated. The amount of single copy genes differ between the two approaches and indicate that 15 to 20% of the genes are still single copy genes (Fig 6).

Overall, the bioinformatic and experimental data indicates that the DortE4 genome contains a large proportion of genome duplication, which is suggestive of a whole genome duplication (WGD) event. The results of the BUSCO analysis and the blast comparison between *D*. *rose* and *M*. *brunnea* indicate that the majority of genes are only duplicated once, which argues against multiple segmental duplications and instead for a single duplication event. Based on the high degree of similarity between the different paralogs we can assume that the duplication has occurred relatively recently.

Such duplications are well studied in yeasts and were first described for *Saccharomyces cerevisiae* [66] but have also been shown for many other species [61, 64, 67]. Both allo- and autopolyploid species are described in the literature.

Reports about WGDs in fungi are not just restricted to yeast, Albertin and Marullo mention in their review of polyploidy in fungi that many other polyploid species occur, e.g. the plant pathogen *Botrytis allii* or the blastocladiomycete *Allomyces macrogynus* mentioned before [66]. In most of the reported cases a WGD event is followed by a loss of duplicated genes [63, 64, 65]. This might be an ongoing process occurring in the *D*. *rosae* genome and is an explanation for the different patterns of the PCR-RFLP analysis of the tested isolates and the 15–20% of genes that show no duplicated in the BUSCO and Blast analyses. Only a comparison of the complete genome sequence of different isolates can give an answer to this question. Here, the isolates R6 and I001 might be of particular interest due to the clear differences to isolate DortE4. In other fungal species WGDs are often connected to changes in ploidy level. But the unimodal distribution of the k-mer distributions (Fig 1, S1 File) and the relatively low level of similarity between the paralogs (Fig 3) argues against this phenomenon in the *D*. *rosae* genome because these dissimilarities exceed the amount of variation typically observed for allelic variation.

Gene duplication and gene-loss are key mechanisms in evolution [68, 69, 70]. A WGD event and the following loss of duplicated genes is in most cases a neutral and non-adaptive process, but it can also result in neofunctionalization or subfunctionalization of duplicated genes, the development of alternative pathways or the remaining of a duplicated gene with redundant function as a genetic buffer [68].

In yeast it is hypothesized that the WGD is a mechanism of diversification, adaption and specialization [66]. In the genome of Microsporidia, which are obligate endoparasitic fungi, it is shown that a WGD event has influenced the local host adaption [71]. For the plant pathogen *Rhizopus oryzae* it was shown that the ancestral WGD has led to an expansion of different gene families that included different virulence factors [65]. It would be interesting to examine if the WGD event influences the *D*. *rosae* genome in similar way. One hint is the increase in virulence factors. The *D*. *rosae* genome contains more than four times as many matches in the PHI base than observed for the genome of the closely related pathogen *M*. *brunnea* [55]. It would be also interesting to analyze how the WGD influences the effector content of the genome. Effector genes are often embedded in highly dynamic regions like TE rich areas or subtelomeric regions, and increase their diversity by rearrangements, duplications, insertions and deletions [6, 72], comparable to the processes occurring during differential gene loss after the genome duplication.

### Concluding remarks

We present here the draft genome of *Diplocarpon rosae*, the causative agent of the blackspot disease on roses. With 2457 scaffolds (>500 bp) the assembly is still fragmented. Nevertheless, it contains almost the complete gene space indicated by analysis with the BUSCO pipeline. Noticeable is the estimated genome size ranging from approximately 70 to 90 Mb depending on the used approaches, which is outstandingly large for a fungal genome. This fact cannot be exclusively explained by its content of TEs and other repetitive elements as it is for other fungi. Based on multiple points of evidence, we propose that a whole genome duplication event occurred relatively recently in the genome of *D*. *rosae*. We could show experimentally that the duplication is neither an artefact of the assembly nor a spontaneous event occurring during *in vitro* culture. There are also indicators that the genome is already in a state of reduction and that different isolates have lost different proportions of the duplicated genome. Whole genome sequence comparison of additional isolates is necessary to clarify this point. Another important question is the influence of the duplication on the pathogenic features of the blackspot fungus. In this regard a more detailed study of the secretome and effector content of the genome is necessary. In conclusion, the *D*. *rosae* genome sequence is a useful tool to study genome duplication outside of the model system yeast, and in the context of plant pathogenesis. This can give new insights into the evolution of pathogenic features and effector proteins, and will be important in understanding the dynamics of this pathogen as the roles of duplicated loci are analyzed functionally.

## Materials and methods

### Fungal isolates

Fungal isolates were multiplied on detached leaves of the susceptible rose cultivar “Pariser Charme” as previously published [73]. They comprise the isolates DortE4 (Dortmund, Germany), R6 (Ahrensburg, Germany), Br2402 (Ahrensburg, Germany), I001 (Lucca, Italy), S003 (Sweden), D005 (Groß Lüsewitz, Germany) [16, 24, 73].

Mycelium of the isolate DortE4, which was used for genome sequencing, was also cultivated *in vitro* on 0.8% agar plates containing 3% liquid biomalt extract (Villa Natura, Kirn, Germany).

### DNA isolation from mycelium and sequencing

Conidia were washed from agar plates and inoculated into 0.5 l of liquid biomalt medium containing 3% liquid biomalt extract in distilled water (Villa Natura, Kirn, Germany). After six weeks of culture at room temperature with moderate shaking (100 rpm) fungal biomass was harvested by centrifugation. The mycelia were ground in liquid nitrogen in a mortar and DNA was extracted with the Biozym (Hessisch Oldendorf, Germany) MasterPure DNA-extraction kit according to the manufacturer’s instructions. Isolated DNA was further purified by one phenol/chloroform extraction and two chloroform extractions, precipitated with isopropanol and subsequently resuspended in TE-buffer and quantified both spectrophotometrically and by gel electrophoresis through 1% agarose gels.

The purified DNA was used for paired-end library preparations with the Epicentre Nextera DNA Library Preparation Kit (Epicentre, Madison, USA) according to the manufacturer’s guidelines. After preparation the library was size selected on a 1% agarose gel and purified using the Qiagen Gel Extraction kit (Hilden, Germany) to produce an average insert length of approximately 500bp. A mate-pair library was prepared with the same DNA using the Nextera Mate-Pair Library Preparation Kit (Illumina, San Diego, USA)) with size-selection (~3 kb) performed on a 0,75% agarose gel and purified using the Qiagen Gel Extraction Kit. The paired-end library was sequenced on an Illumina HiScan SQ with 2x100bp (v3) chemistry. The mate-pair library was sequenced on an Illumina HiSeq 2500 with 2x125bp read length (v4) chemistry.

454 sequence data was generated by Macrogen (Seoul, South Korea), who performed the library preparation and pyrosequencing on a Roche (Basel, Switzerland) 454 GS-FLX system.

### Trimming and assembly

Adapter and quality trimming was performed with the CLC Genomics Workbench (Qiagen, Hilden, Germany). Removal of adaptor content and trimming of poor quality data (<Q20) of the 454 reads was performed with the CLC Genomics Workbench (Qiagen, Hilden, Germany) resulting in an average read length of 440 bp.

The same software was also used for trimming of the Illumina paired-end sequences of the short insert library to a read quality below a PHRED score of Q20 and for trimming of the 3’ ends to a read length of 2x80bp.

Mate-pair data were trimmed using Trimmomatic 0.32 [74] to remove Nextera transposase and TruSeq adaptor sequences as well as poor quality data (<Q20).

The *de novo* assembly algorithm of CLC Genomics Workbench 6.5 (Qiagen, Hilden, Germany) was used to generate a contig-level *de novo* assembly using a combination of short insert library paired-end Illumina and 454 data. The assembly was performed with a “word size” (k-mer size) of 50 and automatic “bubble size”. Contigs were updated after re-mapping reads to the initial assembly and a minimum contig size of 200bp was selected. Scaffolding with paired-end data was performed as part of the CLC *de novo* assembly. Further scaffolding with mate-pair data was performed using Opera-LG (v2) [75]. For Opera scaffolding a ploidy level of 1 was specified and mapping of mate-pair reads to contigs was performed with the BWA software package (version 0.7) [76].

### Gene prediction and annotation

For the gene prediction and structural annotation the MAKER 2.3.1.8 pipeline [35] was used, which combines repeat masking, different prediction tools with evidence based quality control and gene model editing. The repeat masking was done by using RepeatMasker 4.0 [77] with RMBlast search algorithm and the Repbase [78] database. Three different *de novo* prediction tools were combined in the pipeline: the self-training tool GeneMark-ES [37], Augustus [36] with the prediction models of *B*. *cinerea* and SNAP 3 [79] which was trained by three rounds of hint-based MAKER prediction. As evidence for MAKER annotation the predicted proteome of the closely related fungus *M*. *brunnea* [55] and an assembly of the RNA-Seq and MACE data were used. As an additional tool tRNAscan [80] was integrated.

The functional annotation was done with Blast2GO 3.3 [40] and Blastx against the NCBI NR protein database (E-value cutoff e-10). The integrated Gene Ontology (GO) term annotation [81] and InterProScan [41] were both performed using default parameters.

### Additional bioinformatic analysis

To determine the genome size, the tool Jellyfish 1.1.10 [82] was used to produce a k-mer distribution (k-mer size 17, 20, 22, 25, 30, 35) with reads from a small insert library. The data processing was done in R [83]. The genome size was calculated according to procedure used by Li and colleagues for the giant panda sequence [29] and by Liu *et al*. used for the *Brassica oleracea* genome [30] and the GenomScope Software [31] (S1 File).

The repeat structure of the genome was analyzed with the RepeatMasker 4.0.6 software [77] in combination with the RepeatModeler package 1.0.8 [84] and the Repbase [78] database. The cross-match search engine from the Phrap package [85] was applied for this screening. For a more detailed screening for SSR-motifs, the SSR Locator software [56] was applied with a minimum of ten repeats per motif type.

Analyses with the BUSCO pipeline version 1.2 [58] were performed, in genome as well as in transcriptome mode with the fungi dataset.

Different variants of the BLAST+ package [86] were used for all vs all Blast alignments of the paralogous sequences with an E-value cutoff of 1e-10.

For calculation the identity scores of the duplicated BUSCO orthologs a multiple sequence alignment was performed with the mafft 7.3 tool using the FFT-NS-1 alignment algorithm [62]. The resulting alignment was used for generating an identity matrix with the software trimAl 1.2 [87]

### DNA isolation from conidia and PCR-RFLP of paralogous sequences

DNA for this analysis was isolated from conidia that were washed from infected “Pariser Charme” leaves according to the manufacturer’s instructions with the MasterPure DNA-extraction kit (Biozym, Hessisch Oldendorf, Germany). The only divergence from the protocol was the extension of all incubation steps.

The PCR-RFLP analysis was performed for six BUSCO orthologous pairs and six *D*. *rosae* isolates. To distinguish between the two paralogous sequences, specific primer pairs were designed with the Primer3 web tool [88]. PCR reactions were performed in a 20 μl volume containing 10 ng DNA, 0.1 mM dNTP, 0.25 μM forward and reverse primer, 1 U *Taq* polymerase (DCS, DNA cloning service, Hamburg, Germany) and 1x reaction buffer [89]. The following cycling conditions were used: initial denaturation for 10 min at 94°C, followed by 30–35 cycles of 60s at 94°C, 60s at 64°C and 120s at 72°C and a final elongation of 10 min at 72°C.

The PCR reaction was then used for restriction digestion with EcoRI or DraI (Thermo Fisher Scientific, Waltham, USA). Both PCR and restriction digestion products were separated on 1.5% agarose gels.

The amplified fragments of the genes DR003069 and DR002900 of all isolates were sequenced with the Sanger technique. The PCR was performed with the Primestar proof reading *Taq* polymerase (Takara Bio, Clontech, Mountain View, USA) and the above-mentioned amplification protocol. Only the cycling conditions were changed as followed: 98°C for 5 min, 32 cycles of 10 s at 98°C, 15 s at 64°C and 120s at 72°C following the final elongation of 10 min at 72°C. PCR-products were purified with the NucleoSpin Gel and PCR Clean-up kit (Macherey-Nagel, Düren, Germany) following the manufacturer’s instructions. Fragments were ligated into the pJET cloning vector (Thermo Fisher Waltham, USA) using the blunt-end ligation protocol. Sanger sequencing was performed by GATC Biotech (Konstanz, Germany) using the standard pJET sequencing primer.

The resulting sequences from the isolates and the corresponding gene sequences of related fungi were used for generation of a phylogenetic tree using the software MEGA 7.0.18 [90]. The tree was constructed with the Maximum Likelihood method based on the Tamura-Nei model [91]. The percentage of replicate trees in which the associated sequences clustered together in the bootstrap test (500 replicates) are shown next to the branches [92].

### Generation of the expression data

MACE (Massive Analysis of cDNA Ends) data [43, 93] for three time points (0, 24, 72 hpi) and three biological replicates for the compatible interaction of the isolate DortE4 with the susceptible rose variety “Pariser Charme” (PC) were generated so that each of the three biological repeats is derived from an independent inoculation experiment. To expand the analysis, the RNA of the three samples of the 72 hpi time point were also used for a conventional RNA-Seq approach. PC leaves were spray inoculated in a detached leaf assay with a spore concentration of 5x10^5^ spores per ml.

Immediately after sampling, 30 mg leaf material was frozen in liquid nitrogen and RNA was extracted with the Qiagen (Hilden, Germany) RNeasy plant mini kit according to manufacturer’s manual. Library preparation and sequencing was either done by GenXPro (Frankfurt am Main, Germany) for the MACE data or GATC biotech (Konstanz, Germany) for the RNA-Seq data.

Data analyses were performed with the CLC Genomics Workbench 9.0.1 (Qiagen, Hilden, Germany). Quality trimming was done with default parameters. Read mapping for expression profiling was performed with default parameters except the similarity and length fraction which were set to 0.9 respectively 0.95 to increase the sensitivity. A gene was considered to be expressed at a time point if reads were mapped in a least two repeated experiments. Quantitative gene expression levels were not taken into account due to the increasing biomass of the fungus, instead the genes were only classified as expressed or not expressed.

## Supporting information

S1 File Genome size estimation by the k-mer distribution. (DOCX)

S2 File Annotation statistics. (DOCX)

S3 File Blast2Go annotation table. (XLSX)

S4 File Expression data generated with MACE and RNA-Seq. (XLSX)

S5 File Result of the Blast to the Phi database. (XLSX)

S6 File CAZy-annotation. (XLSX)

S7 File RepeatMasker results. (XLSX)

S8 File Results of the BUSCO pipeline. (XLSX)

S9 File Identity values of the duplicated BUSCO orthologs. (XLSX)

S10 File Details about the PCR-RFLP. (DOCX)

S11 File Alignment of the paralogous sequences DR002900 and DR003069. (FAS)

S12 File BlastP to the *M*. *brunnea* proteom. (XLSX)
