# Supplementary material for: A draft genome sequence of the rose black spot fungus Diplocarpon rosae reveals a high degree of genome duplication
Source: PLoS One. 2017 Oct 5;12(10):e0185310. doi: 10.1371/journal.pone.0185310 (PMC5628827; doi:10.1371/journal.pone.0185310)
Supplement: S1 File — (DOCX) [file pone.0185310.s001.docx]

**Supplemental file 1:**

**Genome size estimation by the k-mer distribution**

Three different methods were used to estimate the genome size. All use a k-mer distribution (example Figure 1, main text and Supplemental Figure 1) of a small insert library to estimate the mean k-mer depth.

Li *et al*. are using the formula: N=(M*L)/(L-K+1) to estimate the real sequencing depth (N) from the k-mer coverage (M) and the average read length (L), and the k-mer size (K) [1]. With the given data (Supplementary Table 1) this resulted in a sequencing depth of 37.6. By dividing the total number of sequenced bases with this value we obtained an estimated genome size between 84.99 to 91.36 Mb depending on the chosen k-mer size.

Supplemental Table 1: Genome size estimation according to Li *et al*. [1]

| **k-mer size** | **Average read length** | **k-mer depth** | **Calculated sequencing depth** | **No. of sequenced bases [Gb]** | **Estimated genome size [Mb]** |
| --- | --- | --- | --- | --- | --- |
| 17 | 79.52 | 30 | 37.6 | 3.19 | 84.99 |
| 20 | 79.52 | 28 | 36.8 | 3.19 | 86.76 |
| 22 | 79.52 | 27 | 36.7 | 3.19 | 87.00 |
| 25 | 79.52 | 25 | 35.8 | 3.19 | 89.15 |
| 30 | 79.52 | 23 | 36.2 | 3.19 | 88.17 |
| 35 | 79.52 | 20 | 34.9 | 3.19 | 91.36 |

In the second used method according to Liu *et al*. the genome size G is estimated by the total number of k-mers dived by the k-mer depth [2]. Expressed as a formula: G=(R*(L-K+1)-B)/M were (R) is the total number of sequenced reads, (L) is the average read length, (K) is the k-mer size, (M) is the k-mer coverage and (B) the number of k-mers with a low frequency (<4). The symbol (B) has the function as a corrective factor for sequencing errors. Calculating the genome size with this method resulted in values between 83.61 to 88.60 Mb.

Supplemental Table 2: Genome size estimation according to Liu *et al.* [2]

| **k-mer size** | **Average read length** | **k-mer depth** | **No. of reads [M]** | **No. of low frequent k‑mers [M.]** | **Estimated genome size [Mb]** |
| --- | --- | --- | --- | --- | --- |
| 17 | 79.52 | 30 | 40.14 | 41.57 | 83.61 |
| 20 | 79.52 | 28 | 40.14 | 45.94 | 85.12 |
| 22 | 79.52 | 27 | 40.14 | 48.00 | 85.22 |
| 25 | 79.52 | 25 | 40.14 | 50.56 | 87.12 |
| 30 | 79.52 | 23 | 40.14 | 53.48 | 85.85 |
| 35 | 79.52 | 20 | 40.14 | 55.16 | 88.60 |

The third method estimates the genome size and other parameters based on a mixture model of four different negative binominal distribution implemented into the GenomeScope webserver 1.0 [3]. The software calculates the relative abundance of heterozygous and homozygous, unique and two-copy sequences to estimate the heterozygosity and repeat fraction as well as the error rate. The estimated genome sizes range from 77.54 to 73.53 Mb and a repeat fraction between 29.8% and 41.7% (Supplementary Table 3).

Supplemental Table 3: Genome size estimation with GenomeScope [3]

| **k-mer size** | **Estimated genome size [Mb]** | | **Estimated repeat size [Mb]** | | **Heterozygosity [%]** | | **Read Error Rate [%]** | | **Model Fit [%]** | |
| --- | --- | --- | --- | --- | --- | --- | --- | --- | --- | --- |
|  | min | max | min | max | min | max | min | max | min | max |
| 17 | 72.54 | 72.57 | 30.25 | 30.27 | 0.57 | 0.58 | 0.13 | 0.13 | 97.74 | 99.40 |
| 20 | 72.92 | 72.95 | 27.98 | 27.98 | 0.50 | 0.51 | 0.12 | 0.12 | 97.97 | 99.48 |
| 22 | 73.09 | 73.12 | 26.91 | 26.92 | 0.46 | 0.67 | 0.12 | 0.12 | 98.10 | 99.56 |
| 25 | 73.25 | 73.27 | 25.51 | 25.52 | 0.42 | 0.42 | 0.12 | 0.12 | 98.31 | 99.62 |
| 30 | 73.42 | 73.45 | 23.50 | 23.50 | 0.35 | 0.36 | 0.11 | 0.11 | 98.56 | 99.68 |
| 35 | 73.51 | 73.53 | 21.90 | 21.91 | 0.31 | 0.31 | 0.11 | 0.11 | 98.77 | 99.76 |

| 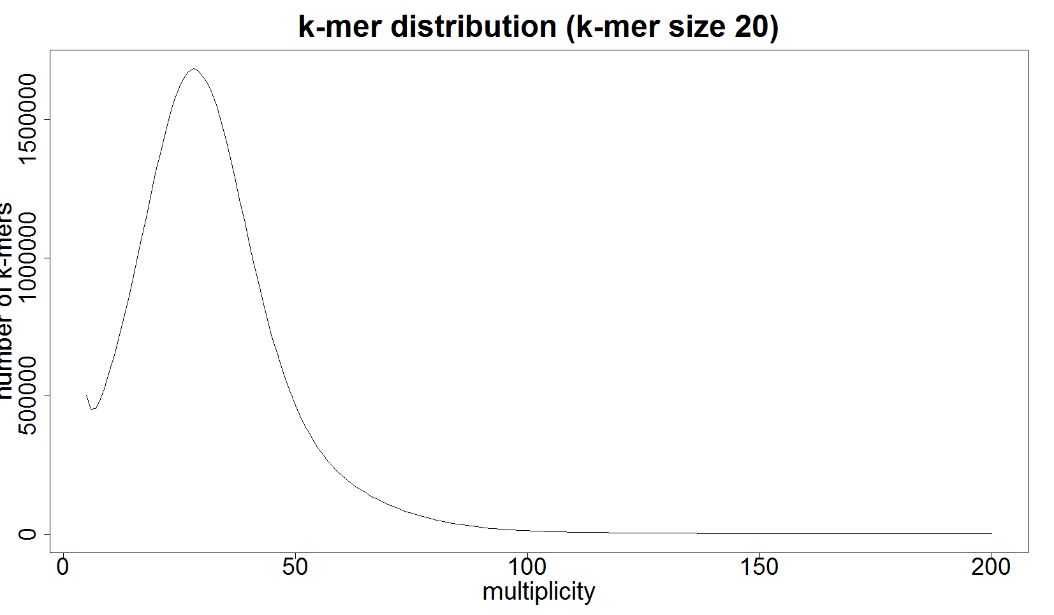  **A** | 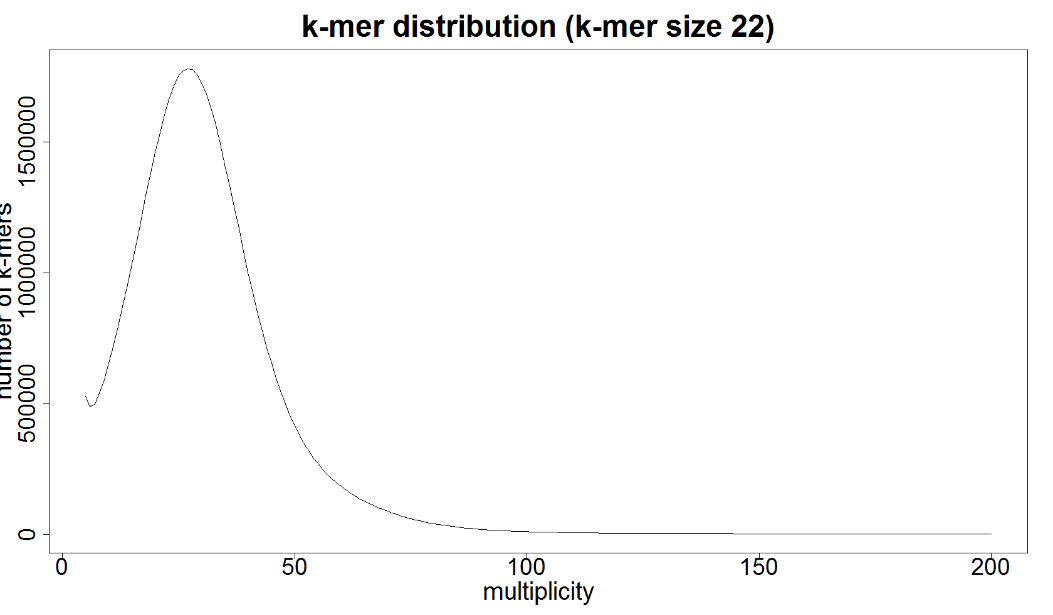  **B**  **D** |
| --- | --- |
| 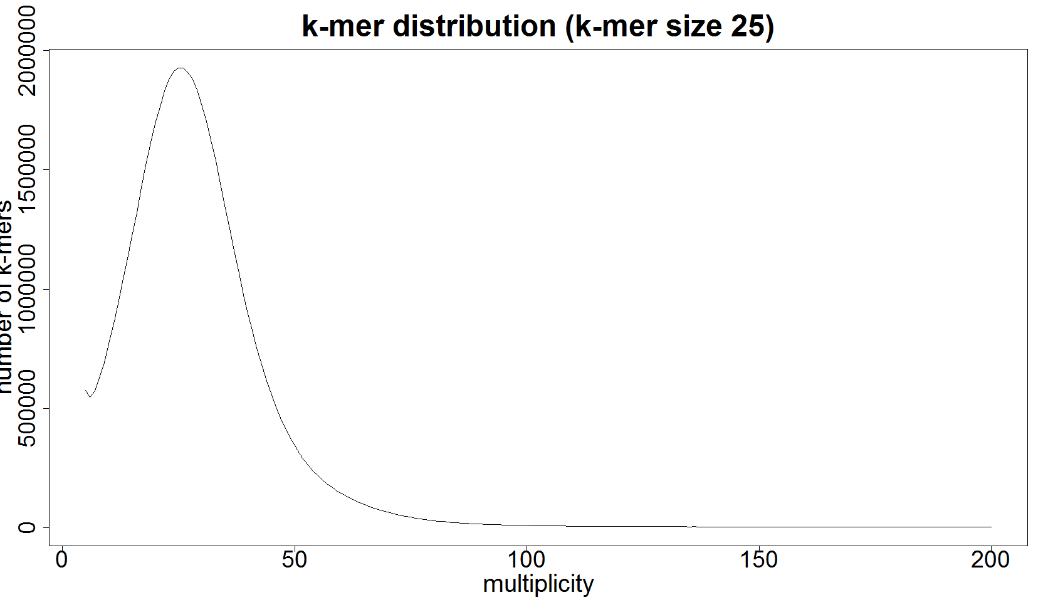  **C** | 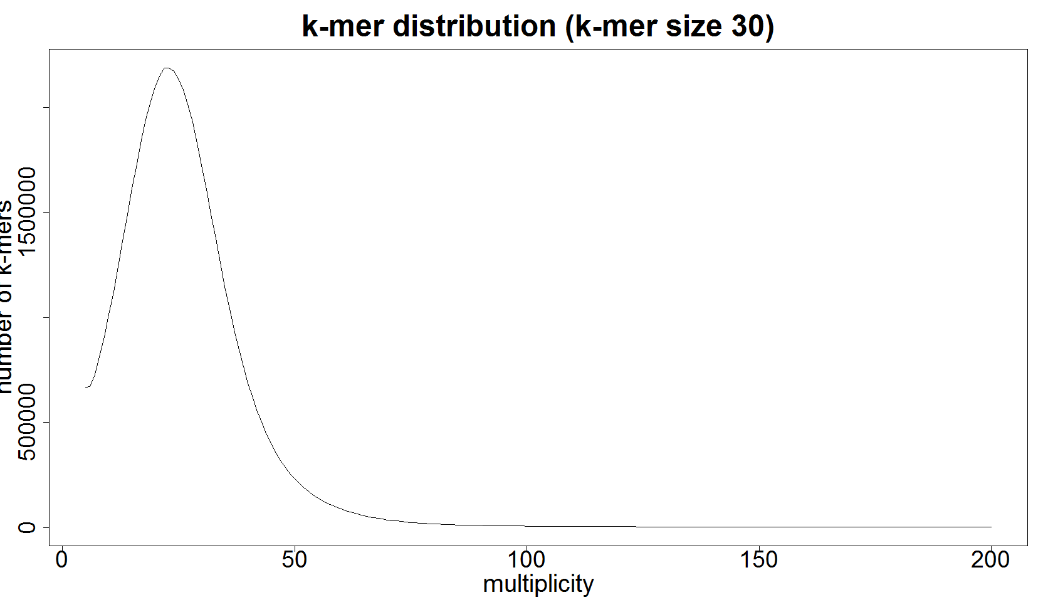 |
| 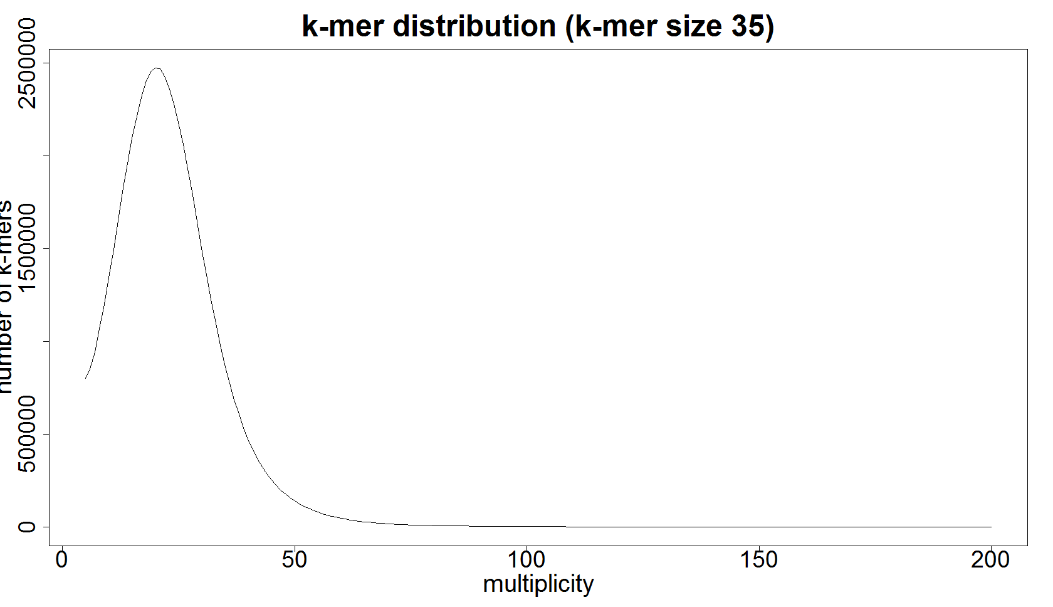  **E** | |

Supplemental Figure 1: k-mer distributions generated with Jellyfish [4] and R [5] based on the small insert Illumina library. The plots display the number of k-mers (A: k-mer size 20, B: k-mer size 22, C: k-mer size 25, D: k-mer size 30, E: k-mer size 35) generated from the reads (y-axis) that occur with a given multiplicity (x-axis). The peak represents the mean k-mer depth. K-mers with extremely low frequency (<5) are not displayed and are considered to contain sequencing errors.

| 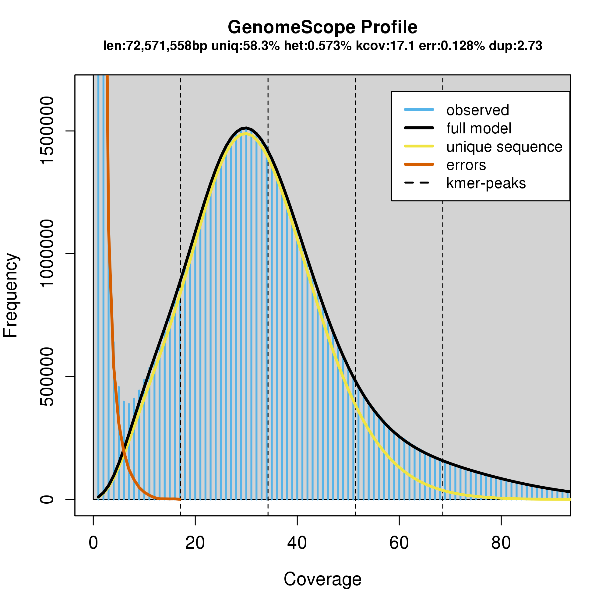  **A** | 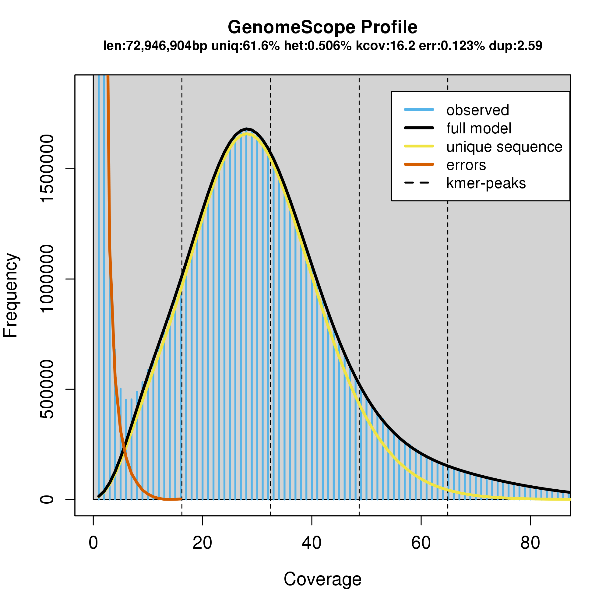  **B** |
| --- | --- |
| 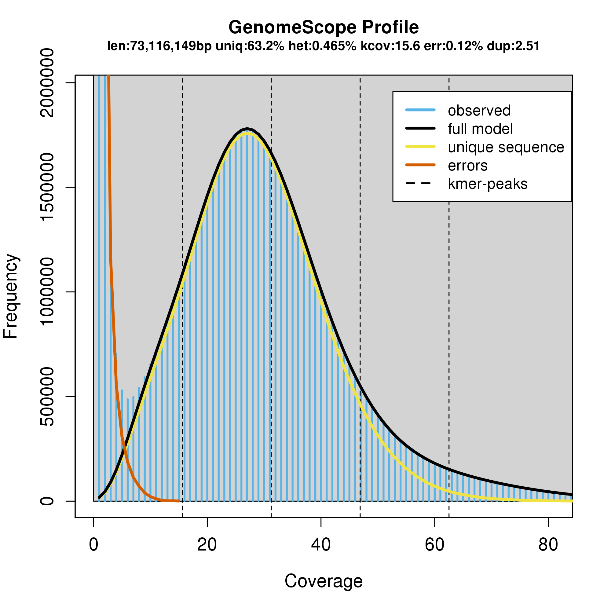  **C** | 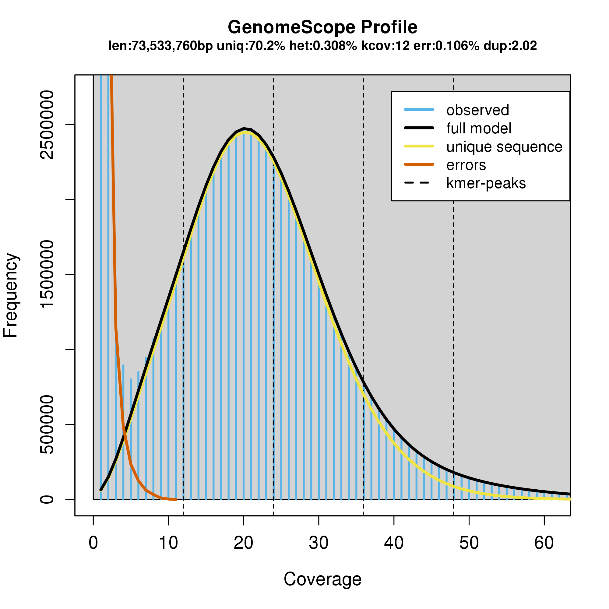  **F** |
| 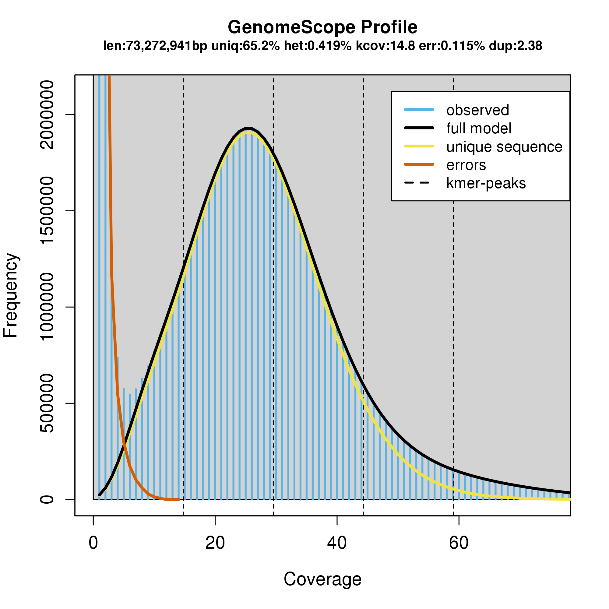  **D** | 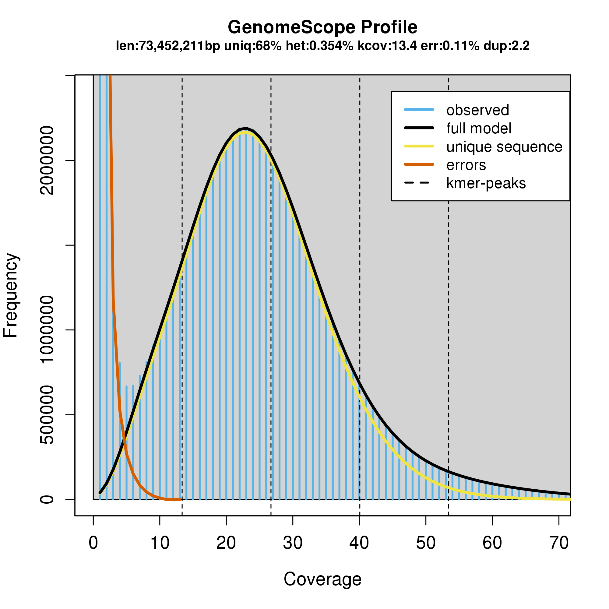  **E** |

Supplemental Figure 2: Analysis of the k-mer distributions generated with Jellyfish [4] with the GenomeScope [3] software to estimate the genome size. The plot displays the number of k-mers generated from a small insert Illumina library (y-axis) that occur with a given multiplicity (x-axis). The plot also contain a graph of the fitted model (black), of the distribution of unique k-mers (yellow) as well as the distribution of potential erroneous k-mers. The dotted vertical line indicates the position of the four peaks the software uses for the model fitting. The different figures depict the distribution of different analysed k-mer sizes: A: k-mer size 17, B: k-mer size 20, C: k-mer size 22, D: k-mer size 25, E: k-mer size 30, F: k-mer size 35.

**References**

1. Li R, Fan W, Tian G, Zhu H, He L, Cai J, *et al*. The sequence and de novo assembly of the giant panda genome. Nature. 2010; 463: 311-317.

2. Liu S, Liu Y, Yang X, Tong C, Edwards D, Parkin IAP, *et al*. The Brassica oleracea genome reveals the asymmetrical evolution of polyploid genomes. Nature Communications. 2014; 5.

3. Vurture G, Sedlazeck F, Nattestad M, Underwood C, Fang H, Gurtowski J, Schatz M.. GenomeScope: fast reference-free genome profiling from short reads. Bioinformatics. 2017; 33(14): 2202-2204.

4. Marcais G, Kingsford C. A fast, lock-free approach for efficient parallel counting of occurrences of k-mers. Bioinformatics. 2011; 27: 764-770.

5. Team RC. R: A Language and Environment for Statistical Computing [https://www.R-project.org/].
