# Supplementary material for: A draft genome sequence of the rose black spot fungus Diplocarpon rosae reveals a high degree of genome duplication
Source: PLoS One. 2017 Oct 5;12(10):e0185310. doi: 10.1371/journal.pone.0185310 (PMC5628827; doi:10.1371/journal.pone.0185310)
Supplement: S2 File — (DOCX) [file pone.0185310.s002.docx]

**Supplemental file 2:**

**Annotation statistics**


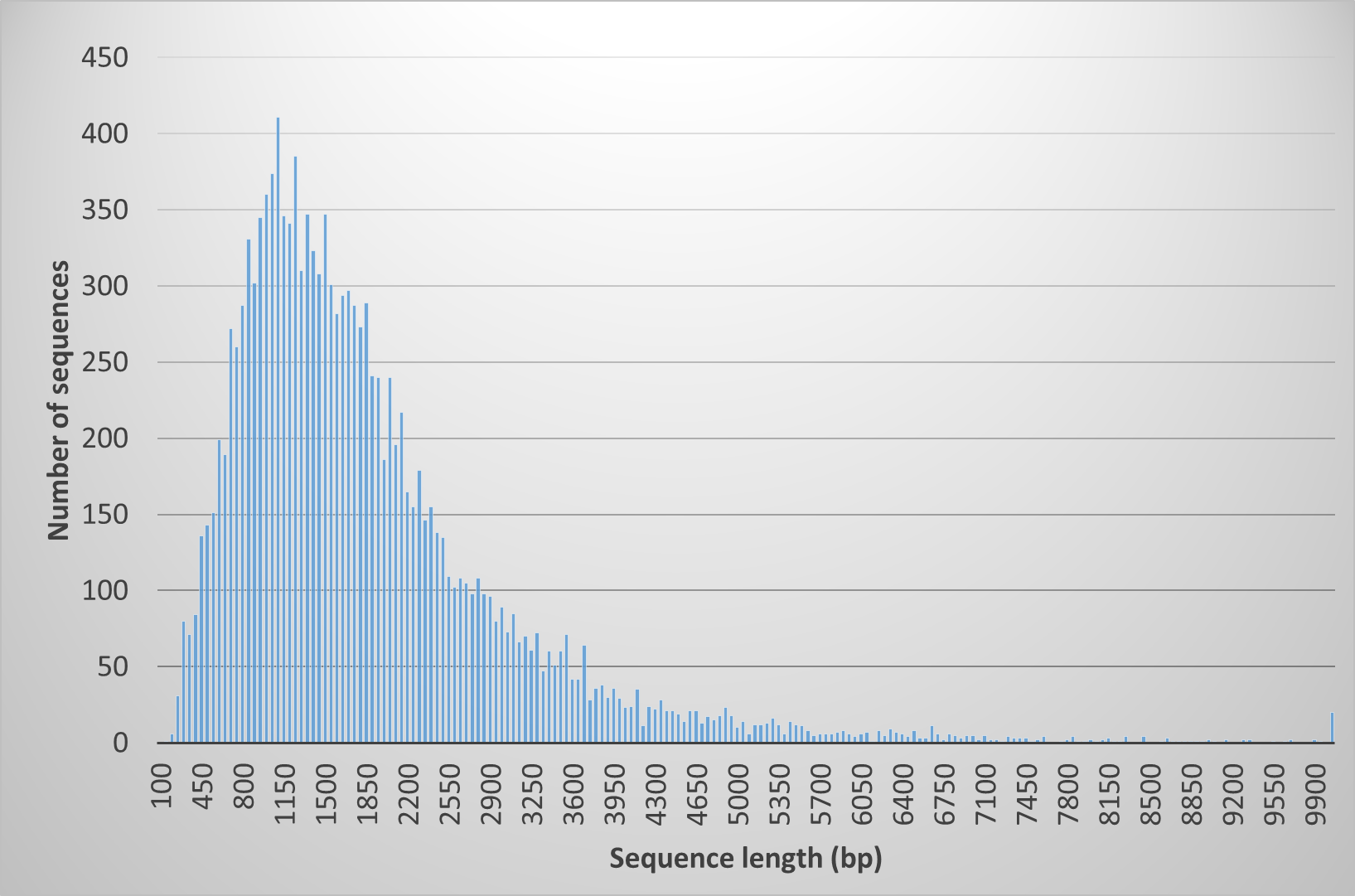


**Supplemental Figure 3: Sequence length distribution of the gene models generated with the Maker pipeline [1].**

**Supplemental Table 4: Descriptive annotation statistics generated with the GAG pipeline [2].**

|  | Annotation statistics |
| --- | --- |
| Total sequence length | 68113174 |
| Number of genes | 14004 |
| Number of mRNAs | 14004 |
| Number of exons | 46930 |
| Number of introns | 32923 |
| Number of CDS | 14007 |
| Overlapping genes | 397 |
| Contained genes | 10 |
| CDS: complete | 13064 |
| CDS: start, no stop | 381 |
| CDS: stop, no start | 411 |
| CDS: no stop, no start | 151 |
| Total gene length | 25968480 |
| Total mRNA length | 25968480 |
| Total exon length | 22862029 |
| Total intron length | 3172297 |
| Total CDS length | 21825303 |
| Shortest gene | 140 |
| Shortest mRNA | 140 |
| Shortest exon | 3 |
| Shortest intron | 4 |
| Shortest CDS | 24 |
| Longest gene | 19700 |
| Longest mRNA | 19700 |
| Longest exon | 14856 |
| Longest intron | 7549 |
| Longest CDS | 18858 |
| mean gene length | 1854 |
| mean mRNA length | 1854 |
| mean exon length | 487 |
| mean intron length | 96 |
| mean CDS length | 1558 |
| % of genome covered by genes | 38.1 |
| % of genome covered by CDS | 32 |
| mean mRNAs per gene | 1 |
| mean exons per mRNA | 3 |
| mean introns per mRNA | 2 |


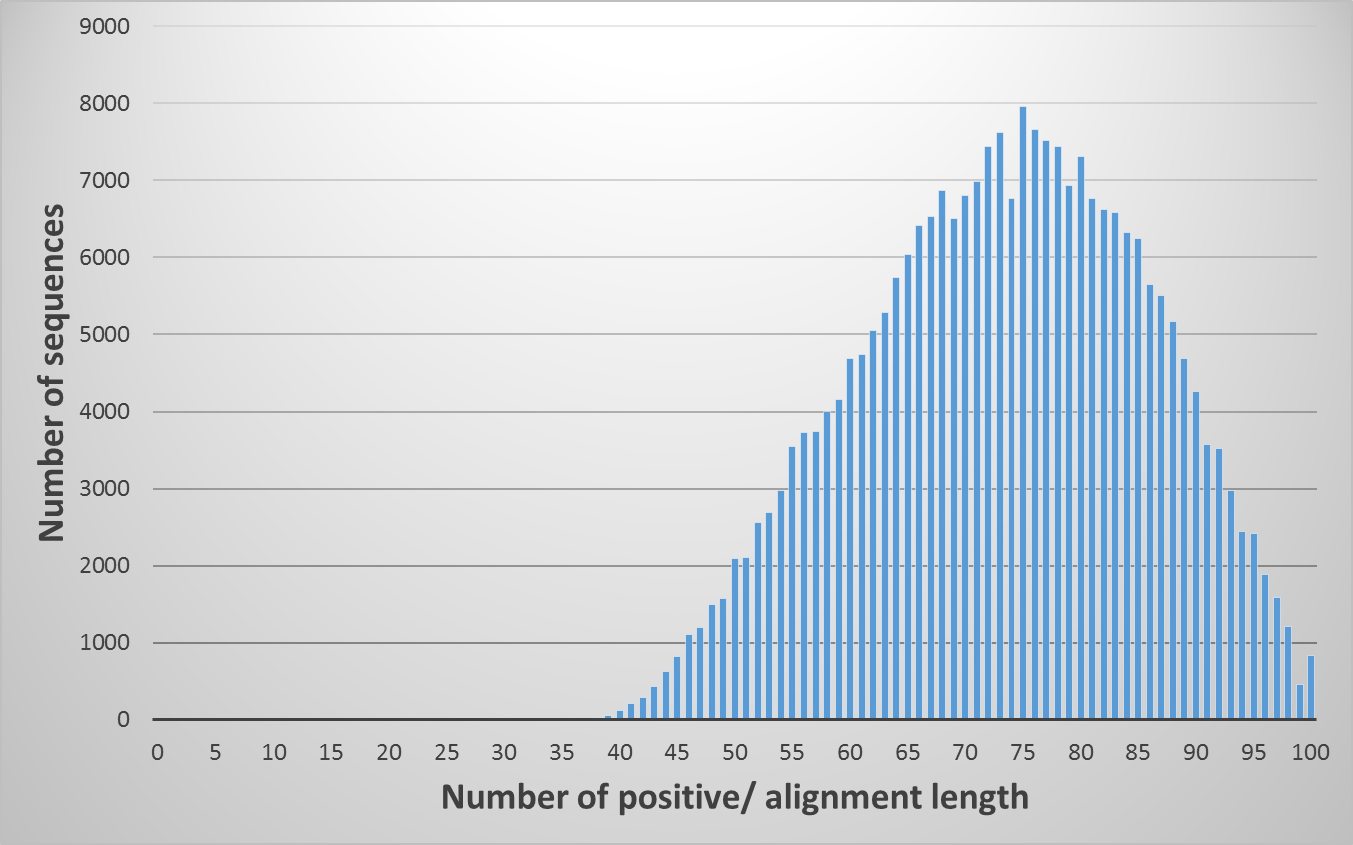


**Supplemental Figure 4: Similarity distribution of all Blast matches generated with the Blast2Go software [3].**


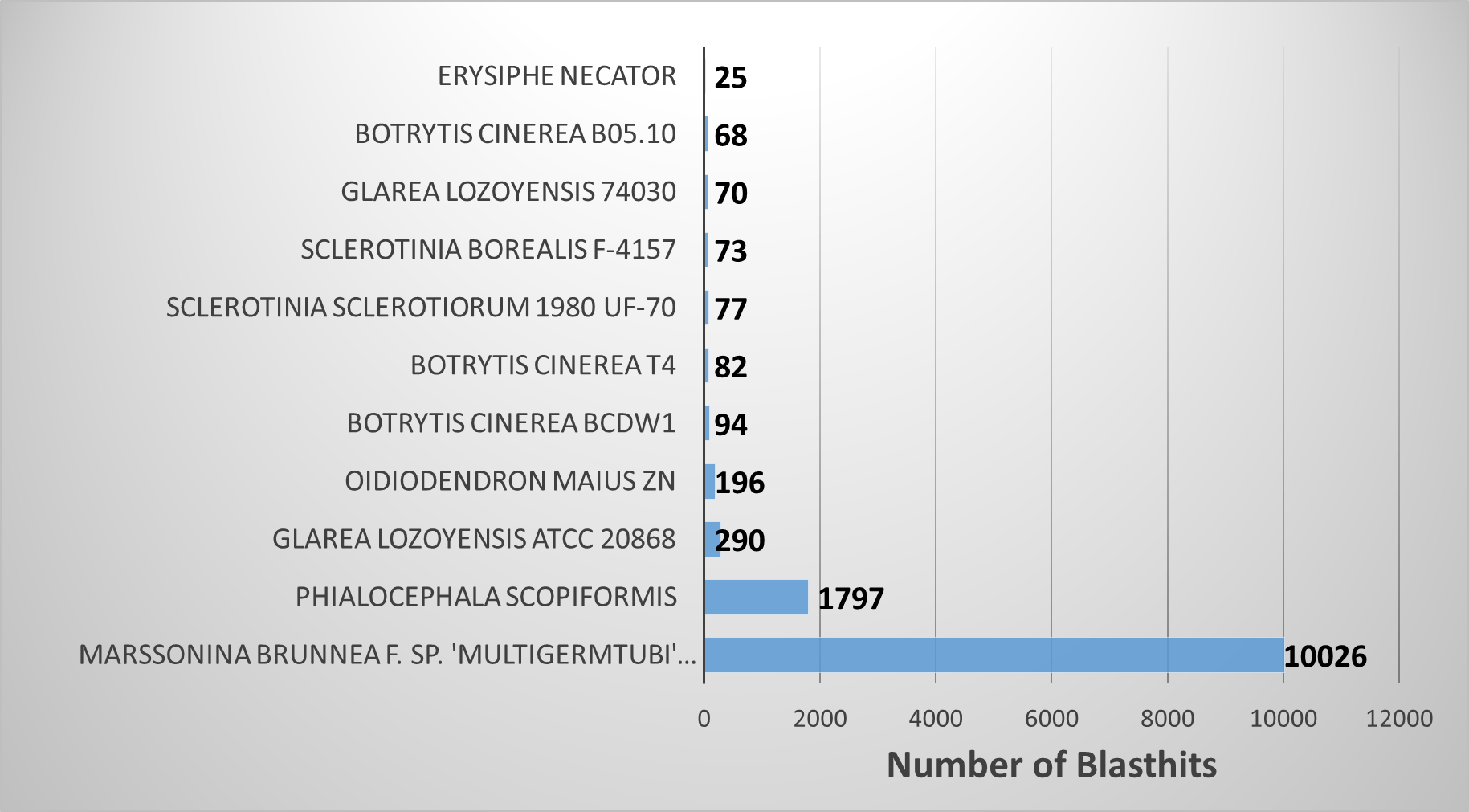


**Supplemental Figure 5: Species distribution of the best Blast matches generated with the Blast2Go software [3].**

**References**

1. Cantarel BL, Korf I, Robb, Sofia M. C., Parra G, Ross E, *et al*. MAKER: An easy-to-use annotation pipeline designed for emerging model organism genomes. Genome Research. 2008; 18:188-196.

2. Hall B., DeRego T., Geib S.. GAG: the Genome Annotation Generator (Version 1.0) (2014); http://genomeannotation.github.io/GAG. Accessed 14. Aug. 2017

3. Conesa A, Gotz S, Garcia-Gomez JM, Terol J, Talon M, et al.Blast2GO: a universal tool for annotation, visualization and analysis in functional genomics research. Bioinformatics. 2005; 21: 3674-3676.
