# Supplementary material for: A draft genome sequence of the rose black spot fungus Diplocarpon rosae reveals a high degree of genome duplication
Source: PLoS One. 2017 Oct 5;12(10):e0185310. doi: 10.1371/journal.pone.0185310 (PMC5628827; doi:10.1371/journal.pone.0185310)
Supplement: S10 File — (DOCX) [file pone.0185310.s010.docx]

**Additional file 8:**

**PCR-RFLP analysis of the genome duplication**

Supplemental Table 5: Overview of the PCR-RFLP to distinguish between different BUSCO homologs within the *D. rosae* genome. The primer pairs are designed to amplify only one of the paralogs. The second feature to differentiate between the sequences is a restriction site that only one of the two fragments contain. The table shows the size of the PCR fragments as well as of the resulting restriction fragments.

| BUSCO | *D. rosae* paralogues | PCR fragment | Restriction  enzyme | Fragment 1 [bp] | Fragment 2 [bp] | Fragment3  [bp] |
| --- | --- | --- | --- | --- | --- | --- |
| BUSCOfEOG7D2FR2 | DR005870 | 2216 | EcoRI | 556 | 1660 |  |
|  | DR011096 | 2177 | EcoRI | 2177 |  |  |
| BUSCOfEOG7K9KD3 | DR004273 | 2043 | EcoRI | 2043 |  |  |
|  | DR002774 | 1987 | EcoRI | 172 | 1815 |  |
| BUSCOfEOG7MD52Z | DR002315 | 1574 | EcoRI | 1574 |  |  |
|  | DR001421 | 1555 | EcoRI | 183 | 1372 |  |
| BUSCOfEOG7R83CB | DR002947 | 1420 | DraI | 150 | 1270 |  |
|  | DR003022 | 1461 | DraI | 1461 |  |  |
| BUSCOfEOG783N6S | DR002900 | 1340 | EcoRI | 323 | 1017 |  |
|  | DR003069 | 1377 | EcoRI | 1377 |  |  |
| BUSCOfEOG7DRJFG | DR008457 | 1392 | EcoRI | 121 | 621 | 650 |
|  | DR005710 | 1387 | EcoRI | 2043 |  |  |

Supplemental Table 6: Primer sequences of the PCR-RFLP analysis.

| **BUSCO** | ***D. rosae* paralogues** | **Forward primer** | **Reverse primer** |
| --- | --- | --- | --- |
| **BUSCOfEOG7D2FR2** | DR005870 | CCTCCGAATCCTCAGATCCC | GTGCTTGCACGAACTCTCTG |
|  | DR011096 | GCCGCCGAGAAGAAAAGAAA | CCCCGAATCTTGACATCCCA |
| **BUSCOfEOG7K9KD3** | DR004273 | ACGGAATCCCAGCTGCTAAT | CTGCAAGTATGGCACCTTCG |
|  | DR002774 | CTGAGAGAGAGGGTGTGGTG | CAATGGCTCAAGGACCAACG |
| **BUSCOfEOG7MD52Z** | DR002315 | AGGGTATGGTTTGTGCCTCT | TCGGTACTGGTATCTTTCCTCC |
|  | DR001421 | CTATGGTCTGTGCCTCGTCT | TCCTCCTGCCAACATTCCAA |
| **BUSCOfEOG7R83CB** | DR002947 | CCAAATGCGGCAAGATGACT | ATTGCAGCGCTTCGTGTTTA |
|  | DR003022 | CTCCGCTGGCCAATCATAAC | TCAGTCACCAAGGCAGTAGG |
| **BUSCOfEOG783N6S** | DR002900 | ACTCAACCGTGCCAAAGATG | ACCAACCTAAGCCACTTCCA |
|  | DR003069 | CCCAGTAAAGATGTGCGAGC | TAGGAGTCACTGGCATGCAA |
| **BUSCOfEOG7DRJFG** | DR008457 | CTGGAGCTGGTGATGGAGAC | AGCCCGGGAGAATATGTAACA |
|  | DR005710 | TGGAGCTGTTGATAGTGACGT | CCATAGTTTTCCTAAAGCCCGG |
